# An integrative network-driven pipeline for systematic identification of lncRNA-associated regulatory network motifs in metastatic melanoma

**DOI:** 10.1186/s12859-020-03656-6

**Published:** 2020-07-23

**Authors:** Nivedita Singh, Martin Eberhardt, Olaf Wolkenhauer, Julio Vera, Shailendra K. Gupta

**Affiliations:** 1 Department of Biochemistry, Babu Banarasi Das University, Faizabad Road, Lucknow, Uttar Pradesh 226028 India; 2 Laboratory of Systems Tumor Immunology, Department of Dermatology, Universitätsklinikum Erlangen and Faculty of Medicine, Friedrich-Alexander University of Erlangen-Nürnberg, Hartmannstr.14, 91052 Erlangen, Germany; 3 Department of Systems Biology and Bioinformatics, University of Rostock, 18059 Rostock, Germany; 4 Chhattisgarh Swami Vivekanand Technical University, Bhilai, Chhattisgarh 491107 India; 5 Stellenbosch Institute for Advanced Study (STIAS), Wallenberg Research Centre at Stellenbosch University, Mostertsdrift, Stellenbosch, 7600 South Africa

**Keywords:** Melanoma, Systems biology, RNA motif, LncRNA, MiRNA, Transcription factor, Network approach, Data integration

## Abstract

**Background:**

Melanoma phenotype and the dynamics underlying its progression are determined by a complex interplay between different types of regulatory molecules. In particular, transcription factors (TFs), microRNAs (miRNAs), and long non-coding RNAs (lncRNAs) interact in layers that coalesce into large molecular interaction networks. Our goal here is to study molecules associated with the cross-talk between various network layers, and their impact on tumor progression.

**Results:**

To elucidate their contribution to disease, we developed an integrative computational pipeline to construct and analyze a melanoma network focusing on lncRNAs, their miRNA and protein targets, miRNA target genes, and TFs regulating miRNAs. In the network, we identified three-node regulatory loops each composed of lncRNA, miRNA, and TF. To prioritize these motifs for their role in melanoma progression, we integrated patient-derived RNAseq dataset from TCGA (SKCM) melanoma cohort, using a weighted multi-objective function. We investigated the expression profile of the top-ranked motifs and used them to classify patients into metastatic and non-metastatic phenotypes.

**Conclusions:**

The results of this study showed that network motif UCA1/AKT1/hsa-miR-125b-1 has the highest prediction accuracy (ACC = 0.88) for discriminating metastatic and non-metastatic melanoma phenotypes. The observation is also confirmed by the progression-free survival analysis where the patient group characterized by the metastatic-type expression profile of the motif suffers a significant reduction in survival. The finding suggests a prognostic value of network motifs for the classification and treatment of melanoma.

## Background

Melanoma is the most severe form of skin cancer. The incidence of melanoma has risen globally with approximately 96,480 new cases to be diagnosed with almost 7230 estimated deaths in 2019 only in the USA (https://www.cancer.org/cancer/melanoma-skin-cancer/about/key-statistics.html#references). It arises from melanocytes, the pigment-producing cells in the basal layer of the epidermis. The progression of normal melanocytes to metastatic melanoma involves a series of histopathological changes, from radial growth to vertical growth followed by metastatic spread to distant sites [1]. Recent advancements in tools and technologies have generated heterogeneous multi-omics data, providing an opportunity to study and understand the concerted aberrations underlying tumor phenotypes [2]. Tumor phenotypes that involve extensive interactions across cell types, at the cellular and tissue levels are particularly suited for network-based approaches. In our previous work, we have discussed several types of complex disease networks comprising both the protein-coding and the non-protein-coding portions of the genome, along with circulatory components (proteins, metabolites, etc.) to assess the risk of developing a metastatic phenotype [3]. Based on this work, our goal here is to study the interplay between molecules in regulatory networks and its implications in tumor progression. Integration of molecules across regulatory layers composed of microRNAs (miRNAs), long non-coding RNAs (lncRNAs), mRNAs, and transcription factors (TFs) provides insights into molecular mechanisms that cannot be understood by analyzing individual disease factors. Moreover, it is also recognized that communication between regulatory layers is a highly non-linear process, and that can be studied using network-based approaches [4].

Over the last decade, several studies demonstrated the role of miRNAs in the context of tumor growth, invasion, and angiogenesis through translational repression or degradation of their respective target mRNAs [5–7]. Similar to miRNAs, lncRNAs are exquisitely regulated, highly diverse in function, and play an important role in tumorigenesis [8]. However, due to diverse modifications at the levels of transcription, post-transcriptional processing, and chromatin remodeling; the mechanistic impact of most lncRNAs remain unknown [9]. One class of lncRNAs acts as sponges through the presentation of excess miRNA binding sites that sequester miRNAs away from mRNAs, thereby inducing de-repression of gene expression. Some lncRNAs function as decoy molecules that regulate gene expression by competitive inhibition of protein function through sequestration [10–12]. Even though the importance of their role is well established in the context of cancer, only a few experimentally supported lncRNA-protein and lncRNA-miRNA associations have been reported. In the present work, we first examined lncRNAs that are associated with tumor progression from non-metastatic to metastatic melanoma phenotypes by regulating molecules from different regulatory layers. Further, we constructed a network by incorporating melanoma-associated lncRNAs, their potential binding partners (miRNAs and proteins), TFs regulating miRNAs, and melanoma-associated genes. We analyzed the integrated network to find molecular signatures associated with the cross-talk between various network layers in the form of regulatory loops (lncRNA-miRNA-TF).

Furthermore, we integrated patient-derived TCGA skin cutaneous melanoma (SKCM) RNAseq dataset, mean-normalized (per gene) across all TCGA cohorts onto the regulatory network. We identified top-ranked motifs based on topological and non-topological properties of their constituting nodes. More specifically, we used the topology parameters degree, betweenness centrality, closeness centrality, and clustering coefficient. The degree parameter corresponds to the number of edges attached to a node. A node with a high degree is often called a ‘hub’ and is known to play a central role in organizing the network. Hub nodes are more likely to be essential than non-hubs because they have more interaction partners and thus have a higher chance to engage in an essential interaction [13]. The betweenness centrality parameter indicates the influence of a node on the control of information flow in the network. Nodes with high betweenness centrality are also called ‘gatekeepers’ and control the communication between different network components [14]. The closeness centrality parameter can be interpreted as a measure of how quickly a node can interact with other nodes of the network. Such central nodes are important because they are easy to reach and belong to the core of the network where the majority of nodes interact quickly [15]. The clustering coefficient parameter shows the degree of clustering of a typical node’s neighborhood. This property describes the local network structure surrounding a node. In integrated networks, clustering is considerably and significantly higher than expected in random networks [16]. Among the non-topological properties, we have used disease pathway association and context-specific expression profiles of the nodes.

From the top-ranked motifs, we identify unique signatures that can be used to identify patients with a metastatic melanoma phenotype. Investigation of downstream molecules regulated by these signatures helps in deciphering key processes responsible for the development of metastatic phenotype. We suggest that the identified lncRNA-associated regulatory network motifs have a prognostic value to assess the likelihood of metastatic progression.

## Results

**Fig. 1:**
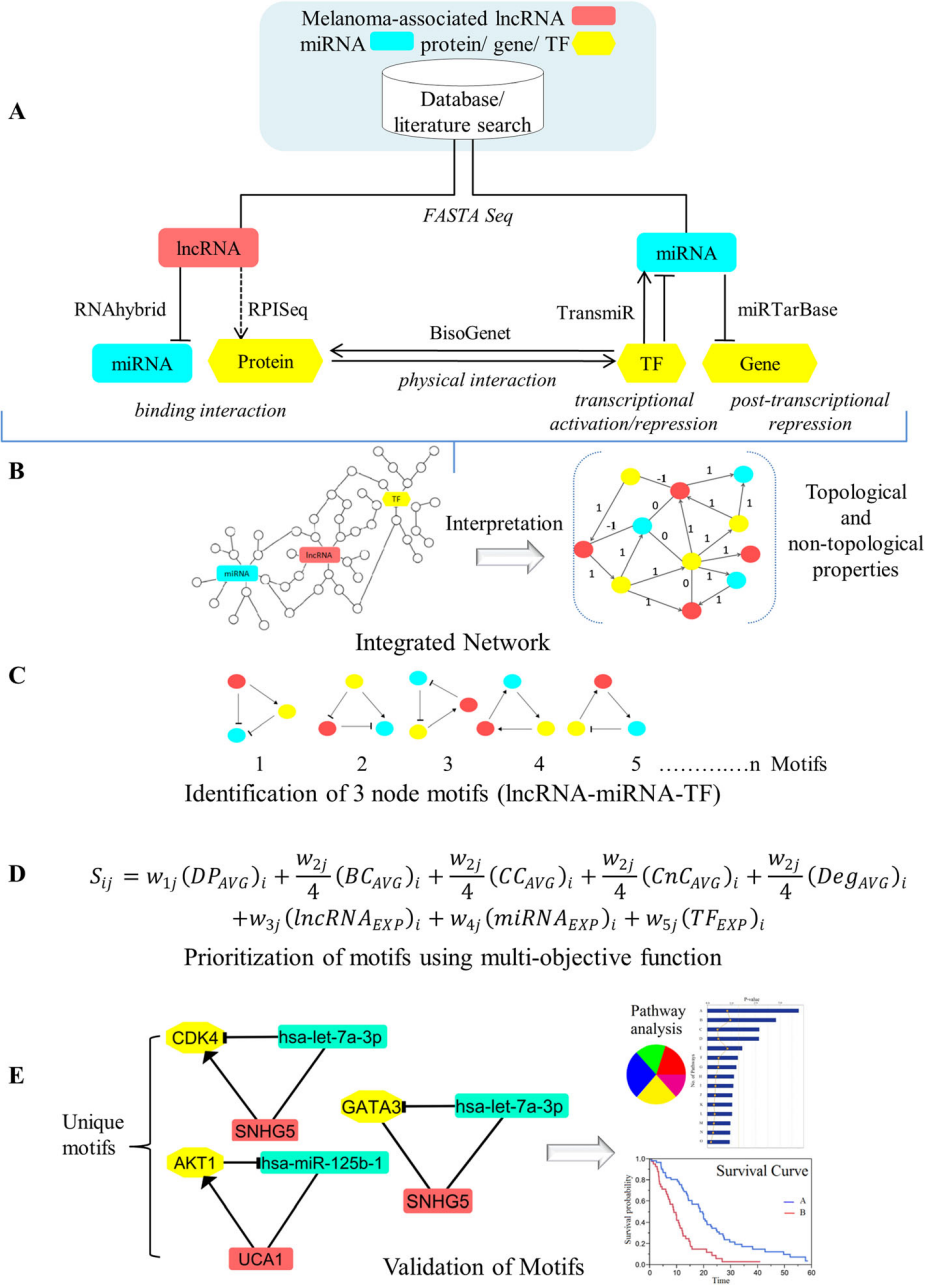
An integrative network-driven pipeline for discriminating non-metastatic and metastatic melanoma phenotypes based on lncRNA-associated regulatory network motifs. **a** Intermolecular interaction data between regulatory molecules (lncRNA, miRNA, and gene/TF) are extracted from public databases, literature, and predicted using existing tools (RNAhybrid, RPISeq). **b** Interactions are merged together to generate an integrated network. Topological properties of the network are investigated with the Network Analyzer plugin in Cytoscape. Non-topological properties, including disease pathway association retrieved from KEGG database and expression profiles of the nodes obtained from a melanoma-specific patient dataset from UCSC Xena. **c** Important regulatory loops comprising of lncRNA-miRNA-TF are predicted with the help of NetDS Cytoscape plugin. **d** Network motifs are prioritized using a multi-objective function by providing user-defined weights in an iterative manner. **e** Calculation of motifs prediction accuracy and survival analysis

**Fig. 2:**
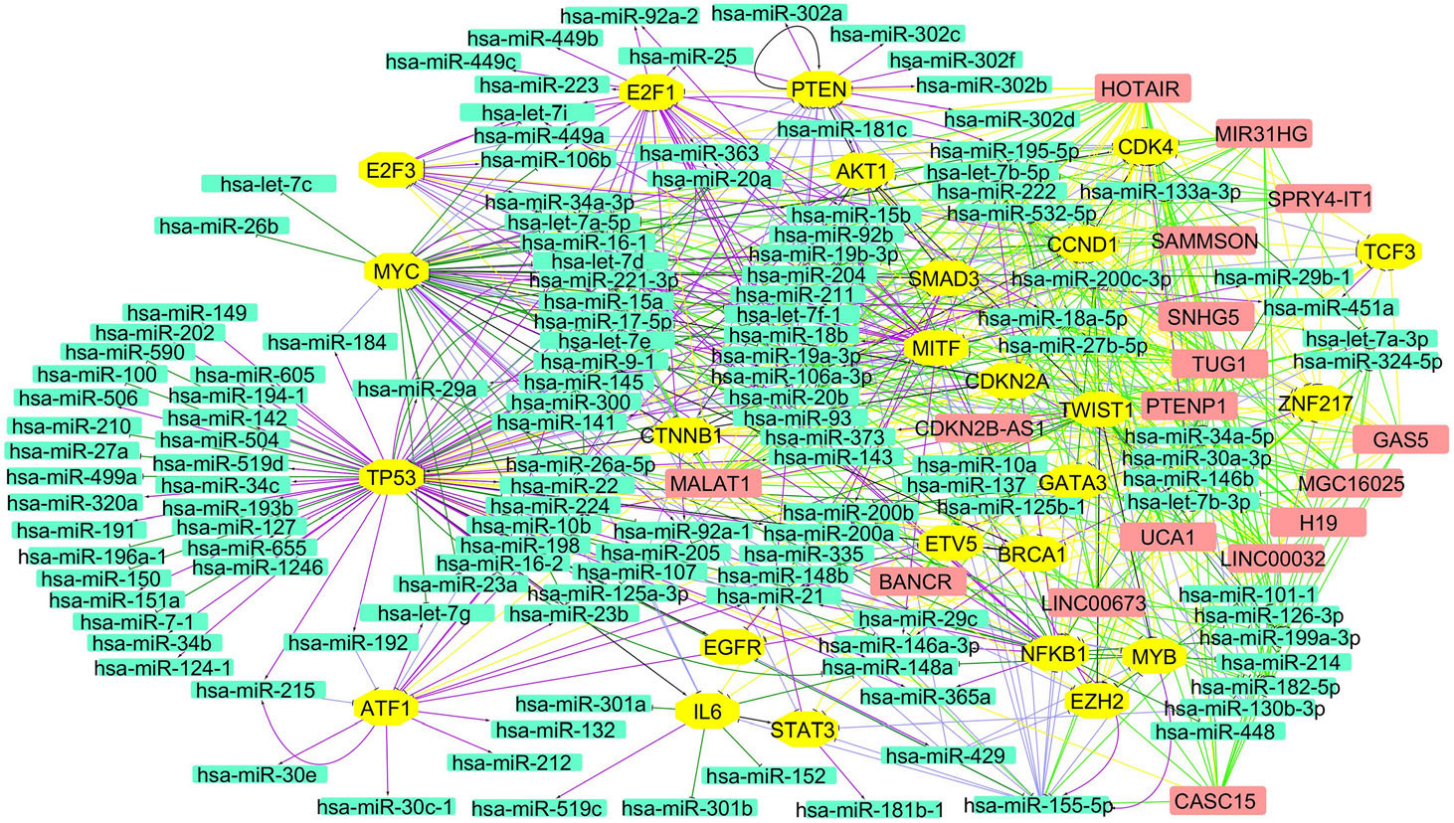
Interaction network of lncRNA, miRNA, and TF in melanoma. Each rectangular node (peach color) indicates lncRNAs which acting as miRNA sponges and affect proteins. Each octagon node (yellow color) indicates experimentally validated TFs which regulate (activation or repression) miRNAs. The network shows miRNAs (rectangular node, cyan color) that negatively regulates the expression of target genes, and it also includes interactions between TFs. The network comprises 174 nodes, including melanoma-associated lncRNAs (17), miRNAs (132), and TFs (25). Network edges (total 655) are colored by the type of interaction between the nodes which are as follows: T bar-shaped edge for functional MTI (light blue color), repression (dark green color), down-regulation (black color); broadhead shaped-arrow for activation (purple color), up-regulation (black color), regulation (purple color); and an undirected edge for binding (fluorescent green color)

The regulatory networks were obtained from a multi-step analysis including the identification of lncRNAs in melanoma, their potential binding partners (miRNAs and proteins), melanoma-associated genes, and TFs regulating miRNAs. The developed pipeline is summarized in Fig. 1. For miRNAs, experimentally validated information related to their target genes is available in several databases. However, for lncRNAs, the information about their interaction partners is largely missing. In our study, we identified miRNAs that can potentially be sponged by melanoma-associated lncRNAs. For that, we built an in-house Python script (Additional file 1: Data S1) to retrieve the nucleotide sequences of melanoma-associated lncRNAs from the NCBI database. We then used the RNAhybrid tool to identify energetically favorable hybridization sites for miRNAs in the target sequence based on dynamic programming. For each of the identified 174 complementary pairs (Additional file 1: Table S1A), we obtained the minimum free energy (mfe) of hybridization and the position of the binding site on the lncRNA. For visualization, hybridization maps of the putative miRNA binding sites on the lncRNAs were generated (Additional file 1: Figures S1-S17). Many miRNAs were found to have either partial or completely overlapping binding sites on the same lncRNA, which may result in binding competition among miRNAs. To identify the miRNA with the highest probability of binding, we assembled miRNA binding clusters from binding sites located in close proximity to each other (distance ≤25 nt) as defined by Saetrom et al. [17]. In each cluster, the miRNA with the most negative mfe of hybridization is selected and accepted into a lncRNA-miRNA interaction network (Additional file 1: Figure S18). Next, we searched miRTarBase for experimentally validated targets (mRNAs) of melanoma-associated miRNAs in *Homo sapiens* at the post-transcriptional level and selected only functional miRNA-target interactions (Additional file 1: Table S1B). The protein products of many of these target genes act as TFs to regulate miRNA precursor gene expression. Here, this translated into a total of 247 TF-miRNA interactions from the TransmiR database as shown in Additional file 1: Table S1C and Figure S19. Furthermore, to connect TFs with the lncRNA layer, we first imported protein sequences from the NCBI Protein database using Python script (Additional file 1: Data S1) and calculated interaction probabilities with the melanoma-associated lncRNAs from Table 1. From the prediction results, we obtained a total of 129 pairs of lncRNAs and TFs which are very likely to interact (Additional file 1: Table S1D). To further identify associations among TFs present in the network, we searched the literature and obtained a total of 22 TF-TF interactions which are reported in Additional file 1: Table S1E. Finally, we constructed an integrated regulatory network of melanoma (in Fig. 2) which includes (i) lncRNA-miRNA; (ii) miRNA-target gene; (iii) TF-miRNA; (iv) lncRNA-TF; and (v) TF-TF interactions. The main purpose of this integrated network is to determine the cross-talk among all the regulatory layers that give rise to the disease phenotype.

**Table 1:** Experimentally validated melanoma-associated lncRNAs in *Homo sapiens*

Melanoma-associated lncRNAs					
LncRNA name	NCBI accession	Alias	Dysfunction Type	Function	PMID
BANCR	NR_047671	LINC00586	Regulation	Cell migration	22581800
CASC15	NR_015410	LINC00340; CANT; lnc-SOX4–1	Regulation	Progression and phenotype Switching	26016895
CDKN2B-AS1	NR_047538	PCAT12; CDKN2B-AS; NCRNA00089; p15AS; ANRIL; CDKN2BAS; CDKN2B-AS	Regulation	Epigenetic silencing	27461581
GAS5	NR_002578	NCRNA00030; SNHG2	Regulation	Cell migration and invasion	26846479
H19	NR_131223	ASM1; WT2; ASM; BWS; LINC00008; NCRNA00008; D11S813E	Expression	Pathogenesis of melasma	19968822
HOTAIR	NR_047528	NCRNA00072; HOXC11-AS1; HOXAS; HOXC-AS4	Regulation	Cell migration and invasion	23862139
LINC00032	NR_026679	C9orf14; NCRNA00032	Mutation	Nevus development	17099875
LINC00673	NR_036488	HILNC75; LUCAIR1; SLNCR1; HI-LNC75; SLNCR; ERRLR01	Expression	Invasion	27210747
MALAT1	NR_002847	NEAT2; LINC00047; NCRNA00047; HCN; PRO2853	Expression	Cell migration	24892958
MGC16025	NR_026664.1	LOC85009; MELOE	Expression	Immunosurveillance	27486971
MIR31HG	NR_027054	LncHIFCAR; hsa-lnc-31	Regulation	Transcriptional regulator	25908244
PTENP1	NR_023917.1	PTEN-rs; PTH2; PTENpg1; PTEN2; psiPTEN	Regulation	Tumor suppressor	21833010
SAMMSON	NR_110000	LINC01212	Regulation	Cell growth and survival	27008969
SNHG5	NR_003038	C6orf160; LINC00044; NCRNA00044; U50HG	Expression	Pathogenesis of metastatic melanoma	26440365
SPRY4-IT1	NR_131221	SPRIGHTLY	Regulation	Cell invasion & proliferation	25344859
TUG1	NR_002321	LINC00080; TI-227H; NCRNA00080	Regulation	Tumor growth and metastasis	29543785
UCA1	NR_015379	LINC00178; UCAT1; CUDR; onco-lncRNA-36; NCRNA00178	Expression	Cell migration	24892958

### Identification of regulatory network motifs composed of lncRNA, miRNA, and TF

For the identification of regulatory network motifs, the integrated network was transformed into a format suitable for the NetDS Cytoscape plugin. Restricting the loop size to three nodes, we obtained 4050 regulatory loops from the integrated network. From this large set, we selected those loops (*n* = 600) that uniquely possess all three types of regulatory components (miRNA, lncRNA, and TF). Further, we used various network topological and non-topological parameters to rank regulatory loops (Additional file 1: Table S1F). The parameters are described in the “Background” section.

### Weighting of parameters for prioritization of motifs

To select the most representative and relevant motifs for the metastatic and non-metastatic group, we prioritized the sets of motifs using a multi-objective function. The function integrates both topological and non-topological parameters, and ranks the motifs. We assigned different weights to the nodes according to Eq. (1) discussed below.

1 $$ {\displaystyle \begin{array}{l}{S}_{ij}={w}_{1j}{\left({DP}_{AVG}\right)}_i+\frac{w_{2j}}{4}{\left({BC}_{AVG}\right)}_i+\frac{w_{2j}}{4}{\left({CC}_{AVG}\right)}_i+\frac{w_{2j}}{4}{\left({CnC}_{AVG}\right)}_i+\frac{w_{2j}}{4}{\left({Deg}_{AVG}\right)}_i\\ {}+{w}_{3j}{\left(\backslash \mathrm{lnc}{RNA}_{EXP}\right)}_i+{w}_{4j}{\left({miRNA}_{EXP}\right)}_i+{w}_{5j}{\left({TF}_{EXP}\right)}_i\end{array}} $$

Where S_*ij*_ is the ranking score of each motif in different weighting scenarios (*i =* 1 … n: motif and *j* = 1 … m: scenario), and *w*_*1j-5j*_ are weighting factors governing the importance of the properties which are: *DP,* motif’s average node disease pathway association i.e. number of motif nodes participating in KEGG pathways (hsa05200-Pathways in cancer, hsa05206–MicroRNAs in cancer, and hsa05202-Transcriptional misregulation in cancer); *BC, motif’s* average node betweenness centrality; *CC,* motif’s average node clustering coefficient; *CnC,* motif’s average node closeness centrality; *Deg,* motif’s average node degree; and *lncRNA*_*EXP*_*, miRNA*_*EXP*_*, TF*_*EXP*_ are metastatic and non-metastatic patient-derived expression profile (pan-cancer normalized log2) of node0, node1, node2 from each motif (*i*) respectively. Weighting scenarios for motif prioritization are given in Additional file 1: Table S2. In the first five scenarios (set 1 and 2), we considered only non-topological parameters while in later scenarios (set 3, 4 and 5) we included combinations of all eligible parameters for motifs ranking. For each weighting scenario, we calculated the result of the objective function for each network motif, and then selected the top ten motifs from each of the 13 weighting scenarios implemented in the multi-objective function. Finally, duplicate motifs were removed, resulting in 20 prioritized motifs in non-metastatic and 25 prioritized motifs in metastatic melanoma (Additional file 1: Table S1G).

### Regulatory connections between nodes of the prioritized motifs

We compared prioritized sets of motifs associated with metastatic and non-metastatic melanoma. We obtained three regulatory network motifs in the metastatic melanoma phenotype whose constituting nodes did not appear in any of the prioritized motifs in case of patients with non-metastatic melanoma.

The first motif features the lncRNA *small nucleolar RNA host gene 5* (SNHG5), which binds to the protein *cyclin-dependent kinase 4* (CDK4). We predicted this interaction using the tool RPISeq, which assigns a high likelihood of binding to this interaction pair (0.979 and 0.75 through SVM and RF classifier, respectively). The expression profile of SNHG5 and its role in facilitating CDK4 expression in tumorigenesis have been defined in the literature [18]. We also found that SNHG5 contains a complementary binding site for the miRNA hsa-let-7a-3p. This implies the sequestration of the miRNA from its target, suggesting that SNHG5 can act as a sponge for the miRNA hsa-let-7a-3p. Next, we obtained a reverse correlation between the expression of miRNA hsa-let-7a-3p and its target protein CDK4, suggesting that CDK4 could also be down-regulated by hsa-let-7a-3p. This interaction was experimentally detected by Kim et al. [19].

The second motif contains the *GATA-binding protein 3* (GATA3), the lncRNA SNHG5, and miRNA hsa-let-7a-3p. The interaction probabilities obtained for GATA3 and SNHG5 are 0.75 (RF) and 0.954 (SVM), respectively. This implies that SNHG5 by binding and sequestering can alter the function of GATA3. It is also apparent that SNHG5 has a sponge effect on miRNA hsa-let-7a-3p as discussed earlier. Further, miRNA hsa-let-7a-3p is predicted to bind to the target site on the GATA3 transcript and negatively regulate the expression of GATA3 protein. This interaction was examined in breast cancer through HITS-CLIP performed by Pillai et al. [20].

In the third motif, comparatively a high-probability pairing was obtained between the lncRNA urothelial cancer-associated 1 (UCA1) and the protein AKT Serine/Threonine Kinase 1 (AKT1), which is higher than first and second motif (0.973 and 0.9 through SVM and RF classifier, respectively). The expression of UCA1 is positively correlated with AKT1 activity and this interaction was experimentally confirmed by Yang et al. [21]. Further, UCA1 down-regulates miRNA hsa-miR-125b-1 by sequestration, serving as a sponge with high-degree complementarity at the binding sites. In turn, AKT1 negatively regulates miRNA hsa-miR-125b-1 at the transcriptional level [22].

### Validation of the prioritized motifs in predicting non-metastatic and metastatic phenotype

As the obtained three motifs were prioritized in the metastatic melanoma phenotype, we hypothesized that these motif signatures can be used to distinguish metastatic melanoma patients from others. To validate our hypothesis, we investigated the expression profile of lncRNA, miRNA, and TF using RNAseq data from 477 TCGA (SKCM) samples available at https://gdc.xenahubs.net version 08-07-2019 [23]. Out of 477 patient samples, we found 408 samples with expression profiles of the nodes constituting all the identified regulatory network motifs.

**Fig. 3:**
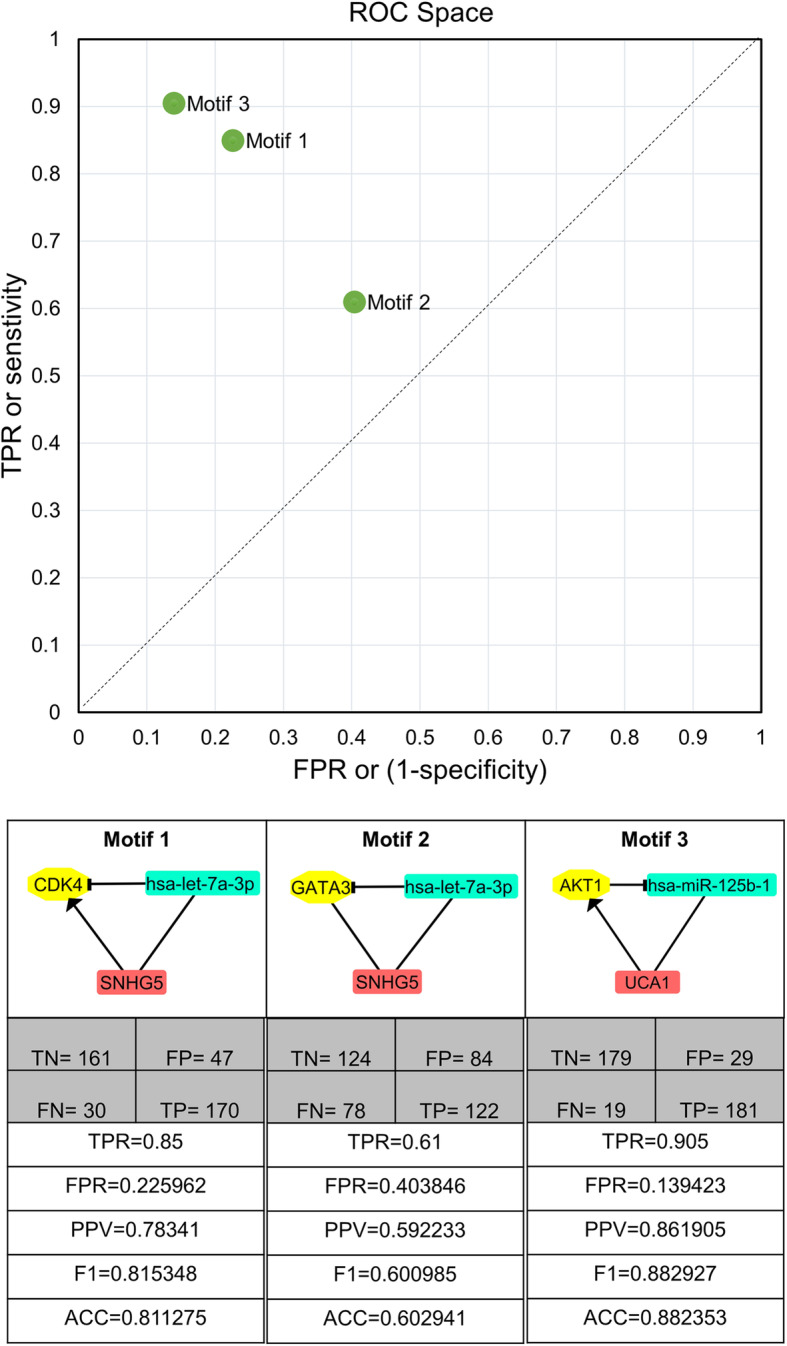
The ROC space of the three motif signature-based classifiers for their ability to discriminate metastatic from non-metastatic melanoma samples. The graph shows the superiority of motif 3 in both sensitivity and specificity. The contingency tables are given below the motifs (True positives TP, True negatives TN, False positives FP, and False negatives FN), followed by additional performance metrics (Precision PPV, F1 measure, and Accuracy ACC) for the three classifiers

First, we grouped patient samples (total 408) into metastatic and non-metastatic phenotype based on their clinical *pathologic_stages*. For that, we classified tumor samples belonging to stages 0, I, IA, IB, II, IIA, IIB, IIC as non-metastatic; and stages III, IIIA, IIIB, IIIC, IV as metastatic [24]. The assignment of melanoma tumor stage generally requires a large number of clinical parameters (such as serum LDH level, the mitotic rate per mm^2^, ulceration status, level of invasion, metastatic volume, number of nodal metastasis, tumor thickness, etc.) and is highly critical to decide therapy regime. To evaluate whether the three unique regulatory network motifs identified in the metastatic melanoma phenotype can help to distinguish metastatic patients, we classified patients based on the motifs’ expression patterns (up-regulation/down-regulation of nodes) (Table 2). For each node, we obtained the canonical expression pattern (up-regulation or down-regulation) in metastatic melanoma from literature. This allowed us to define the motifs’ signature patterns in metastatic samples. For each sample, we then calculated the fold change of each motif node with respect to its overall-mean expression in all 408 samples (Additional file 1: Table S3). The 408 samples were then classified as metastatic if the respective genes’ up−/down-regulation profile matched the motif in question’s signature pattern, or as non-metastatic otherwise (Additional file 1: Table S1H-J). With this classification scheme, the highest prediction accuracy was reached by motif 3 (ACC = 0.88), followed by motif 1 (ACC = 0.81) and motif 2 (ACC = 0.60) as represented in Fig. 3. Overall, the results indicate that motif 3 (UCA1/AKT1/hsa-miR-125b-1) has the best predictive power in distinguishing metastatic and non-metastatic phenotypes of SKCM tumor samples.

**Table 2:** The expression pattern (up-regulation or down-regulation) of motif nodes in Signature and Anti-Signature

Variable	Signature	Anti-Signature
Motif 1	CDK4 (UP)	CDK4 (DOWN)
	SNHG5 (UP)	SNHG5 (DOWN)
	hsa-let-7a-3p (DOWN)	hsa-let-7a-3p (UP)
Motif 2	GATA3 (DOWN)	GATA3 (UP)
	SNHG5 (UP)	SNHG5 (DOWN)
	hsa-let-7a-3p (DOWN)	hsa-let-7a-3p (UP)
Motif 3	AKT1 (UP)	AKT1 (DOWN)
	UCA1 (UP)	UCA1 (DOWN)
	hsa-miR-125b-1 (DOWN)	hsa-miR-125b-1 (UP)

**Fig. 4:**
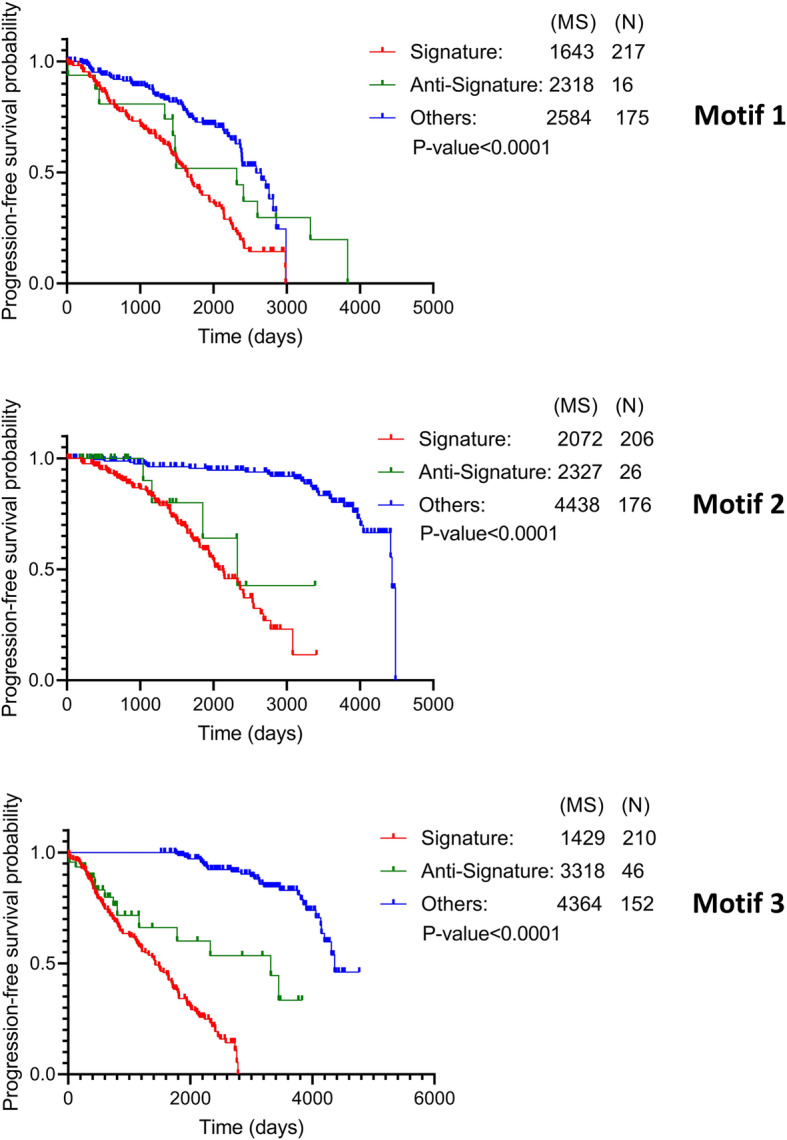
Kaplan–Meier plots for progression-free survival probability of patient subgroups (Signature, Anti-Signature, and Others). This is based on the expression pattern of motif-constituting nodes as defined in Table 2. Patients that do not follow either Signature or Anti-Signature expression patterns are added in Others group. For each group, the number of patients (N) and mean progression-free survival time (MS) in days is provided. The log-rank test was used to assess differences in survival time between all three patient subgroups

Furthermore, we used data from the previous dataset and performed survival analysis considering progression-free survival time using GraphPad Prism 7.05 [25]. For this, we stratified the cohort into three subgroups; 1) samples conforming to the signature expression patterns; 2) samples conforming to the anti-signature expression patterns (i.e. expression pattern opposite to the signature in Table 2); and 3) samples that do not conform to either signature or anti-signature patterns. The log-rank (Mantel-Cox) test was used to compare the survival distributions. We observed a significant correlation between the three subgroups regarding motif signatures and progression-free survival time (Fig. 4). The results obtained from the overall comparison indicated that all the three motifs can distinguish metastatic melanoma patients with a significant time difference (at *P*-value< 0.0001). We also observed that motif 3 showed the largest survival time difference (1889 days) between Signature and Anti-Signature, followed by motif 1 (675 days) and motif 2 (255 days), which is in accordance with our earlier findings in the ROC analysis. Further, motif 3 was best able to differentiate the three patient subgroups in a pairwise fashion (Signature vs Anti-Signature, *P*-value = 0.0019 and Signature vs Others, *P*-value< 0.0001; in Additional file 1: Table S1K). Altogether, our analysis suggested that the expression profiles of nodes in motif 3 (UCA1/AKT1/hsa-miR-125b-1) can be used for quick assignment of metastatic or non-metastatic phenotypes to melanoma patients.

## Discussion

In this article, we study the interplay of molecules (lncRNAs, miRNAs, and TFs) and their integration across regulatory layers of networks to decipher tumor phenotypes and the underlying mechanisms of melanoma metastasis. A network-driven pipeline is developed which combines heterogeneous genomic datasets related to lncRNAs in melanoma, their potential binding partners (lncRNA-miRNA; lncRNA-TF), melanoma-associated genes (miRNA-target gene), TFs regulating miRNAs (TF-miRNA), and TF-TF interactions to determine the cross-talk between various network layers, and their impact on tumor progression and disease phenotype. The study exclusively identified three lncRNA-associated regulatory network motifs in metastatic patients based on the calculation and prioritization of topological and non-topological properties. The approach was evaluated by investigating the expression profile of the motifs and used them to classify patients into metastatic and non-metastatic phenotypes. The prediction accuracy is calculated for each motif through ROC analysis. Subsequently, the method was applied to study three subgroups of patients (Signature, Anti-Signature, and Others) and performed a survival analysis considering progression-free survival time. The results suggested a prognostic value of motif 3 (UCA1/AKT1/hsa-miR-125b-1) for discriminating metastatic and non-metastatic melanoma phenotypes with a high prediction accuracy (ACC = 0.88). It is also confirmed from the observation that the expression profile of motif 3 clearly distinguishes patients with metastatic melanoma phenotype by the lowest mean survival time of 1429 days (at *P*-value< 0.0001).

**Fig. 5:**
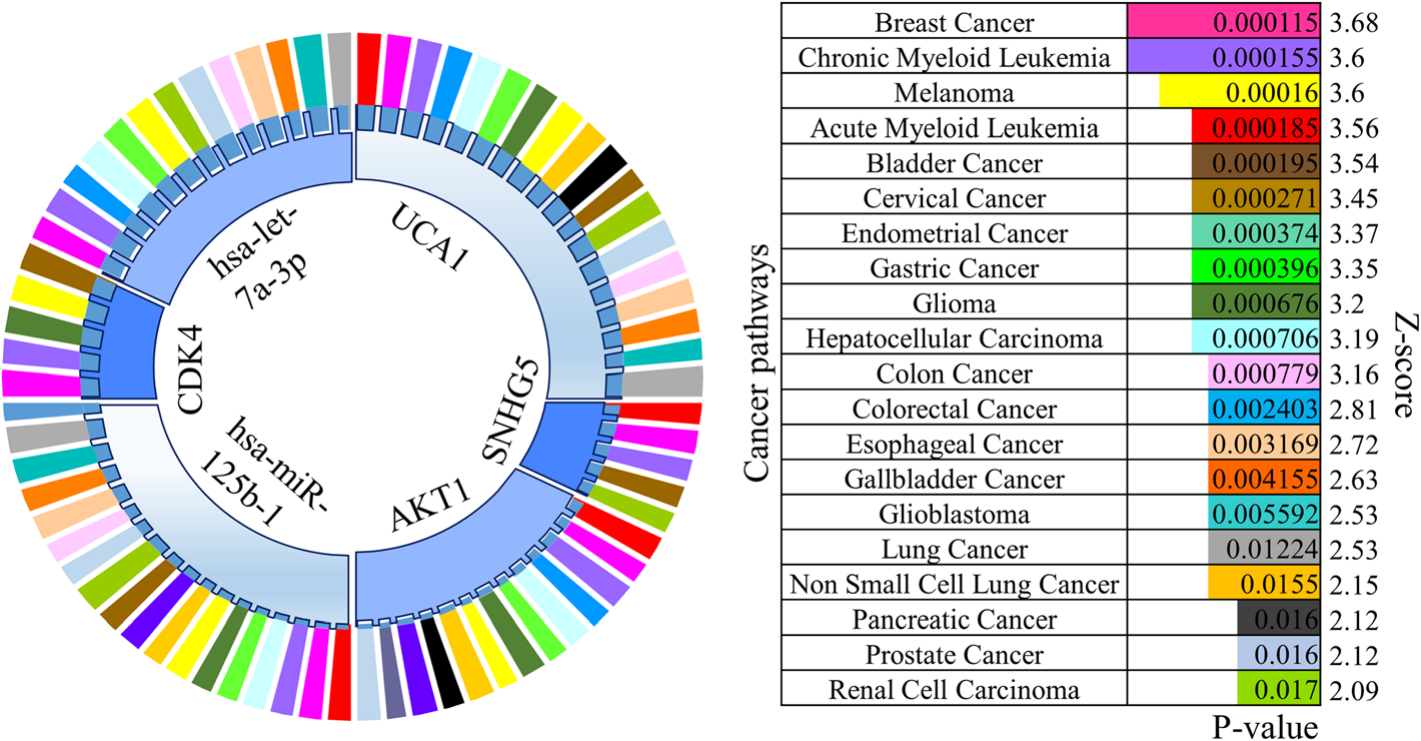
Functional pathway analysis of the constituting nodes of motif 1 and 3 (hsa-miR-125b-1, hsa-let-7a-3p, SNHG5, UCA1, AKT1, and CDK4) showing the involvement of each node over categories of cancer pathways in a doughnut chart. The bar graph shows the top 20 significant cancer pathways sorted by *P*-value< 0.05 and Z-score > 1.65. The length of the bar represents the significance of that specific pathway

Further, to identify the role of the factors in motifs with good predictive power (CDK4/SNHG5/hsa-let-7a-3p and UCA1/AKT1/hsa-miR-125b-1) in the regulation of various metastatic tumors, we used Target Mine web server [26] and selected BH method (Benjamini-Hochberg) for P-value adjustment. For miRNA functional association with tumors, we used TAM 2.0 [27] to compare the queries (hsa-let-7a-3p, hsa-miR-125b-1) with the reference miRNA sets and inferred their disease associations. The results are plotted in a doughnut chart and a bar graph (Fig. 5). From the functional analysis, we identified pathways in which the constituents of the identified motifs participate. The obtained top 20 significant pathways are cancer-associated, especially the ‘breast cancer’ and ‘chronic myeloid leukemia’ pathways are found to be enriched with most of the regulatory molecules of the motifs. This indicates the possibility that the screened motifs are also associated with increased risk for developing metastatic breast cancer and chronic myeloid leukemia. Furthermore, UCA1 and hsa-miR-125b-1 participation were found in 90% of cancer pathways which might suggests that they play a role not only in melanoma progression but also in other metastatic tumors.

There are some limitations to our approach. First, regarding the detailed annotations of the lncRNAs, miRNAs, TFs, and their molecular associations; we observed a poor overlap in the results derived from the different databases and tools. This is probably due to different data resources or algorithms used to predict these interactions. Second, our major focus in this study is on lncRNAs and their interacting miRNA, and TF partners. However, several other relevant interactions can take place in different contexts and may influence the outcome. Hence, a comprehensive view of interactions between lncRNAs and miRNAs or TFs is still required. Third, in cases where the direction of regulation (activation/repression) between biomolecules is not reported in the literature, we have taken as ‘0’ such as lncRNA-miRNA interactions and few lncRNA-TF interactions. So, here the experimental observations’ confirming the nature of their association is missing. Lastly, the expression profile of motifs investigated from RNAseq data are limited by false positives and false negatives. To overcome this, we applied adj. *P*-value< 0.05 for differential screening to control the FP and FN errors.

## Conclusions

The study considers melanoma as an integrated system of regulatory molecules rather than an outcome of isolated molecular events. Our integrated pipeline applies network-based approaches to identify key regulatory components (lncRNA, miRNA, and TF) of the network which enabled a deeper investigation into tumor initiation and progression. Generalizing this pipeline to other datasets would significantly help in the identification of phenotype-based predictive factors for other disease models. The result of the present study reveals multi-level interactions between regulatory layers of a melanoma network can be accessed at https://vcells.net/miRNAs-and-lncRNAs-in-melanoma/. We have uncovered a potential role for the regulatory network motif UCA1/AKT1/hsa-miR-125b-1 in melanoma. There is an 88% chance that the expression profile of the motif will correctly distinguish a patient phenotype as non-metastatic or metastatic melanoma. The result of survival analysis also indicates that the motif has a good discriminating ability. The findings provide more insights into a systems level understanding of melanoma progression that comes through the cross-talk between lncRNA, TF, and miRNA regulatory layers.

## Methods

We used computational strategies that combine heterogeneous genomic data to identify potential coding (TFs) and non-coding (lncRNAs and miRNAs) genes associated with the biogenesis, development, and differentiation of melanoma cells. Figure 1 provides a schematic representation of the developed pipeline.

### Data collection

We obtained experimentally validated melanoma-associated lncRNAs (in *Homo sapiens*) from databases such as LncRNADisease [28], Lnc2Cancer [29], and EVlncRNAs [30], and manually curated them for their functions and associated aliases (in Table 1). Similarly, we derived experimentally validated melanoma-associated miRNAs (in *Homo sapiens*) from databases MiR2Disease [31] and miRBase [32]. Experimentally validated melanoma-associated gene targets of mature miRNAs were collected from miRTarBase [33]. Further, FASTA sequences of lncRNAs and proteins were retrieved from NCBI (https://www.ncbi.nlm.nih.gov) in batch fashion using a Python script.

### Prediction of miRNAs and proteins that interacts with lncRNAs

Sequence complementarity between lncRNAs and miRNAs was assessed using RNAhybrid [34]. The parameters selected for analysis included an upper energy threshold of − 15 kcal/ mole and a restriction to the top 25 human interactors per lncRNA. The *P*-value and minimum free energy (mfe) of hybridization were calculated for hybrid structures. The regulatory direction of binding interactions between lncRNA and miRNA is taken as ‘0’. Since miRNA suppresses target genes, all the regulatory directions were represented as inhibitory ‘-1’. Subsequently, we investigated the proteins which are associated with melanoma and checked their interactions with lncRNAs from Table 1 using the RNA-Protein interaction prediction (RPISeq) tool [35]. The tool provides sequence-based predictions based on Support Vector Machine (SVM) and Random Forest (RF) classifiers trained on RPI2241 and RPI369, datasets of RNA-protein interactions. We set a probability value > 0.5 for positive interactions, and selected threshold values by taking the median predicted values of the positive interactions for both classifiers (i.e. SVM ≥ 0.95 and RF ≥ 0.75). We also searched for the NPInter 3.0 database for registered lncRNA-TF interactions [36]. Regulatory direction from lncRNA to TF was manually searched from literature either as activation ‘+1’ or repression ‘-1’. For those cases where the appropriate regulatory role of lncRNA on TF is missing in the scientific literature, we considered the direction of interaction as binding ‘0’. In addition, we collected transcriptional regulatory information between miRNAs and melanoma-associated proteins which act as TFs from the TransmiR database [37]. In this case, the regulatory direction was defined based on the ‘Action Type’ parameter from the TransmiR database. We considered activation and regulation as ‘+1’, while repression is encoded by ‘-1’. Lastly, we derived TF-TF interactions using the Bisogenet Cytoscape plugin [38]. The regulatory direction between TF-TF was manually obtained from the published literature.

### Network construction and analysis

The regulatory relationships were constructed and visualized using Cytoscape 3.7.0 (https://cytoscape.org/) [39]. Various topological properties including centrality parameters (degree, closeness centrality, betweenness centrality, and clustering coefficient) were calculated for each node using the Network Analyzer plugin in Cytoscape [40].

### Motif finding and survival analysis

Regulatory network motifs were identified in the integrated network using the NetDS Cytoscape plugin [41]. To identify the most relevant motifs for the non-metastatic and metastatic melanoma phenotypes, we followed a method proposed in Khan et al. in 2017 [42]. The method requires the (i) calculation of topological properties of the nodes constituting a motif, (ii) motif-disease pathway associations, (iii) assignment of a differential expression value for a motif based on the change in expression values of the constituent nodes, and (iv) using a weighted multi-objective function as shown in Eq. (1) to rank important motifs. More details of the pseudo code to reproduce the result are given in Additional file 1: Data S2. To identify key molecular signatures from the top-ranked motifs, we first divided patient samples into two groups (i.e. metastatic and non-metastatic melanoma phenotypes) based on their respective clinical stages. Further, we analyzed the expression profile of nodes associated with regulatory network motifs (i.e. up-regulation or down-regulation from their overall mean expression value calculated using all the melanoma patient samples). Patients, where the nodes expression profile (i.e. up-regulation or down-regulation) matches to the signature patterns, are reclassified as metastatic patients, whilst others are assigned to non-metastatic group. This reclassification of patients were compared to the melanoma phenotype based on the clinical *pathologic_stages* to calculate the prediction accuracy of motifs. Analysis of data was performed using MedCalc Statistical Software v14.8 [43]. Later, the top-ranked motifs are analyzed for progression-free survival probability using Kaplan-Meier survival analysis.

### Web interface for visualizing and analyzing the network

To facilitate exploration of the reconstructed network and the analyzed data for interested parties, we uploaded them to the web platform for visualization of biochemical networks vCells https://vcells.net/miRNAs-and-lncRNAs-in-melanoma/. The uploaded network was annotated with additional identifiers for genes, miRNAs, and lncRNAs to allow quick access to external databases. The vCells platform provides tools to project data on top of the molecules in the network, e.g. for expression or differential expression, and to extract sub networks of interest. The network itself is also offered as a downloadable file.

## Supplementary information

**Additional file 1: Table S1A.** Predicted lncRNA-miRNA interactions; **Table S1B.** MiRNA-target gene interactions; **Table S1C.** TF-miRNA interactions; **Table S1D.** Predicted lncRNA-TF interactions; **Table S1E.** TF-TF interactions; **Table S1F.** Topological and non-topological parameters calculated for each node of regulatory network motif; **Table S1G.** Prioritized motifs for metastatic and non-metastatic melanoma phenotype; **Table S1H.** Predictive statistics for motif 1; **Table S1I.** Predictive statistics for motif 2; **Table S1J.** Predictive statistics for motif 3; **Table S1K.***P*-value identified from pairwise and overall comparison of three patient subgroups**; Table S2.** Weighting scenarios for ranking of motifs; **Table S3.** Patient-derived RNAseq expression profile (pan-cancer normalized log 2) of nodes in three prioritized motifs (lncRNA/miRNA/TF); **Figures S1-S17.** Hybridization maps of putative miRNAs binding sites across lncRNA sequences; **Figure S18.** LncRNA-miRNA interaction network. Rectangular nodes designate lncRNA (peach color) and miRNA (cyan color). The network consists of 47 nodes (including 17 lncRNAs and 30 miRNAs) and 174 prioritized edges link the pairs of lncRNA and miRNAs in cluster; **Figure S19.** TF-miRNA interaction network. Experimentally validated target genes of miRNAs which act as TFs are represented by octagon nodes (yellow color) and miRNAs are showed by rectangular nodes (cyan color). The network is comprised of 146 nodes with 25 TFs and 121 miRNAs. The edges of the network (total 247) signify predictions of miRNA regulation by TFs. Arrow-headed lines are for activation (purple color) and bar-headed lines are for repression (green color); **Data S1.** Python script for retrieval of FASTA sequences from NCBI; **Data S2.** Pseudo code for ranking of network motifs.

## Data Availability

The reconstructed network of melanoma is publicly accessible at https://vcells.net/miRNAs-and-lncRNAs-in-melanoma/. FASTA files of lncRNAs and proteins used in this study are available in the NCBI repository https://www.ncbi.nlm.nih.gov/. The accession numbers of lncRNAs are listed in Table 1 and GI numbers of proteins are included in Supplementary Table S1B. Mature miRNA sequences are obtained from miRBase [32]. All data generated and analysed during this study are included in this research article and its supplementary information files.

## Abbreviations

lncRNA: Long non-coding RNA
miRNA: microRNA
TF: Transcription factor
TCGA: The Cancer Genome Atlas
SKCM: Skin Cutaneous Melanoma
KEGG: Kyoto Encyclopedia of Genes and Genomes
NCBI: National Center for Biotechnology Information
SVM: Support Vector Machine
RF: Random Forest
TAM: Tool for Annotations of miRNAs
BANCR: BRAF-activated non-protein coding RNA
CASC15: Cancer susceptibility 15
CDKN2B-AS1: CDKN2B antisense RNA 1
GAS5: Growth arrest specific 5
H19: Long intergenic non-protein coding RNA 8
HOTAIR: HOX transcript antisense RNA
LINC00032: Long intergenic non-protein coding RNA 32
LINC00673: Long intergenic non-protein coding RNA 673
MALAT1: Metastasis associated lung adenocarcinoma transcript 1
MIR31HG: MIR31 host gene
PTENP1: Phosphatase and tensin homolog pseudo gene 1
SAMMSON: Survival associated mitochondrial melanoma specific oncogenic non-coding RNA
SNHG5: Small nucleolar RNA host gene 5
SPRY4-IT1: SPRY4 intronic transcript 1
TUG1: Taurine up-regulated 1
UCA1: Urothelial cancer associated 1
CDK4: Cyclin dependent kinase 4
GATA3: GATA binding protein 3
AKT1: AKT serine/threonine kinase 1
MTI: miRNA-target gene interaction
