# Supplementary material for: An integrative network-driven pipeline for systematic identification of lncRNA-associated regulatory network motifs in metastatic melanoma
Source: BMC Bioinformatics. 2020 Jul 23;21:329. doi: 10.1186/s12859-020-03656-6 (PMC7376740; doi:10.1186/s12859-020-03656-6)
Supplement: Supplementary file 1 — Additional file 1: Table S1A. Predicted lncRNA-miRNA interactions; Table S1B. MiRNA-target gene interactions; Table S1C. TF-miRNA interactions; Table S1D. Predicted lncRNA-TF interactions; Table S1E. TF-TF interactions; Table S1F. Topological and non-topological parameters calculated for each node of regulatory network motif; Table S1G. Prioritized motifs for metastatic and non-metastatic melanoma phenotype; Table S1H. Predictive statistics for motif 1; Table S1I. Predictive statistics for motif 2; Table S1J. Predictive statistics for motif 3; Table S1K.P-value identified from pairwise and overall comparison of three patient subgroups; Table S2. Weighting scenarios for ranking of motifs; Table S3. Patient-derived RNAseq expression profile (pan-cancer normalized log 2) of nodes in three prioritized motifs (lncRNA/miRNA/TF); Figures S1-S17. Hybridization maps of putative miRNAs binding sites across lncRNA sequences; Figure S18. LncRNA-miRNA interaction network. Rectangular nodes designate lncRNA (peach color) and miRNA (cyan color). The network consists of 47 nodes (including 17 lncRNAs and 30 miRNAs) and 174 prioritized edges link the pairs of lncRNA and miRNAs in cluster; Figure S19. TF-miRNA interaction network. Experimentally validated target genes of miRNAs which act as TFs are represented by octagon nodes (yellow color) and miRNAs are showed by rectangular nodes (cyan color). The network is comprised of 146 nodes with 25 TFs and 121 miRNAs. The edges of the network (total 247) signify predictions of miRNA regulation by TFs. Arrow-headed lines are for activation (purple color) and bar-headed lines are for repression (green color); Data S1. Python script for retrieval of FASTA sequences from NCBI; Data S2. Pseudo code for ranking of network motifs. [file 12859_2020_3656_MOESM1_ESM.zip › Data S1-S2.pdf]

**Data S1: Python Script for retrieval of FASTA sequences from NCBI.**

```
from Bio import Entrez
import time
import sys
import subprocess
Entrez.email ="user defined"
## We instead upload the list of ID beforehand
gis=[570359610...] \\ GenInfo identifier, "gi" is the unique sequence record processed by NCBI.
request = Entrez.epost("nucleotide",id=",".join(map(str,gis))) \\ Posts a file containing a list of UIs for
future use in the user's environment to use with subsequent search strategies.
result = Entrez.read(request) \\ This function parses an XML file created by NCBI's Entrez
Utilities, returning a multilevel data structure of Python lists and dictionaries.
webEnv = result["WebEnv"]
queryKey = result["QueryKey"]
handle = Entrez.efetch(db="nucleotide",retmode="xml", webenv=webEnv, query_key=queryKey)
\\ EFetch retrieves records in the requested format from a list of one or more UIs or from
user's environment.
for r in Entrez.parse(handle): \\ This function parses an XML file created by NCBI's Entrez Utilities,
returning a multilevel data structure of Python lists and dictionaries. 'parse' is a generator function that
returns the records one by one. This function is useful for parsing large files.
    # Grab the GI
    try:
        gi=int([x for x in r['GBSeq_other-seqids'] if "gi" in x][0].split("|")[1])
    except ValueError:
        gi=None
    print (">GI ",gi," "+r["GBSeq_primary-accession"]+"
"+r["GBSeq_definition"]+"\n"+r["GBSeq_sequence"][0:100000000000])
with open("output.txt", "w+") as output:
    subprocess.call(["python", "./demo.py"],
    stdout=output); sys.stdout.close()
```

**Data S2: Pseudo code for ranking of network motifs.**

**Algorithm**: prioritization of network motifs

---

**Input:** Mixed class molecules (lncRNA, miRNA, TF) in Node0, Node1, Node2; DP is the number of motif nodes participated in KEGG pathways; BC is betweenness centrality of a node in network; CC is clustering coefficient of a node in network; CnC is closeness centrality of a node in network; Deg is the degree of a node in a network; ExpN0 is expression profile of node0; ExpN1 is expression profile of node1; ExpN2 is expression profile of node2; Sij is the ranking score of each motif in different weighting scenarios (i= 1…n: motif and j = 1…n: scenario); w1j-5j are weighting factors.

**Output:** Resulting set of prioritized motifs

```
1.  motif_groups ← [ ]
2.  for each mixed class data point a in Node0, Node1, Node2 do
3.          if (Node0 = lncRNA && Node1= miRNA && Node2 = TF)
4.          Append Node0, Node1, Node2, DP, BC, CC, CnC, Deg, ExpN0, ExpN1,
    ExpN2          to motif_groups
5.  end
6.  motif_ranking ← [ ]
7.  for each motif in motif_groups do
8.          for i ←1 to no_of_motif
9.                  for j ← 1 to no_of_scenario
10.                         if j<=5 then
11.                             w1 & w2 =0 && sum(w3)+sum(w4)+sum(w5)=1
12.                         if j>=6 && j<=12 then
13.                             if w1=0
14.                                 sum(w2)+sum(w3)+sum(w4)+sum(w5)=1
15.                             else
16.                                 sum(w1)+sum(w3)+sum(w4)+sum(w5)=1
17.                         else
18.                            sum(w1)+sum(w2)+sum(w3)+sum(w4)+sum(w5)==1
19.                        Sij=(w1ij)Normalize(DP)+(w2ij/4)Normalize(BC+CC+CnC+
                           Deg)+(w3ij)Normalize(ExpN0)+(w4ij)Normalize(ExpN1)
                           +(w5ij)Normalize(ExpN2)
20.         Append motif_ranking score for each motif
21. end
22. for each motif in motif_ ranking do
23.         for i ←1 to 10
24. Print motif_ ranking[i]
25. end
```
