# Supplementary material for: An integrative network-driven pipeline for systematic identification of lncRNA-associated regulatory network motifs in metastatic melanoma
Source: BMC Bioinformatics. 2020 Jul 23;21:329. doi: 10.1186/s12859-020-03656-6 (PMC7376740; doi:10.1186/s12859-020-03656-6)

Additional file 1: Figure S1-S17

Hybridization maps of putative miRNAs binding sites across lncRNA sequences: S1, MGC16025map; S2, SPRY4-IT1map; S3, BANCRmap; S4, SAMMSONmap; S5, CDKN2B-AS1map; S6, LINC00673map; S7, MIR31HGmap; S8, CASC15map; S9, SNHG5map; S10, UCA1map; S11, HOTAIRmap; S12, PTENP1map; S13, TUG1map; S14, LINC00032map; S15, MALAT1map; S16, GAS5map; and S17 H19map. The mfe (kcal/mole) values are represented on maps by color-coded boxes: 15-30 (blue), 30-40 (green) and > 40 (red). The miRNA binding clusters are assembled from binding sites in close proximity (≤ 25nt) of each other and indicated in boxes (black). For each cluster, the miRNA with the most negative mfe value of hybridization is underlined.

(Figure S1) **MGC16025map**

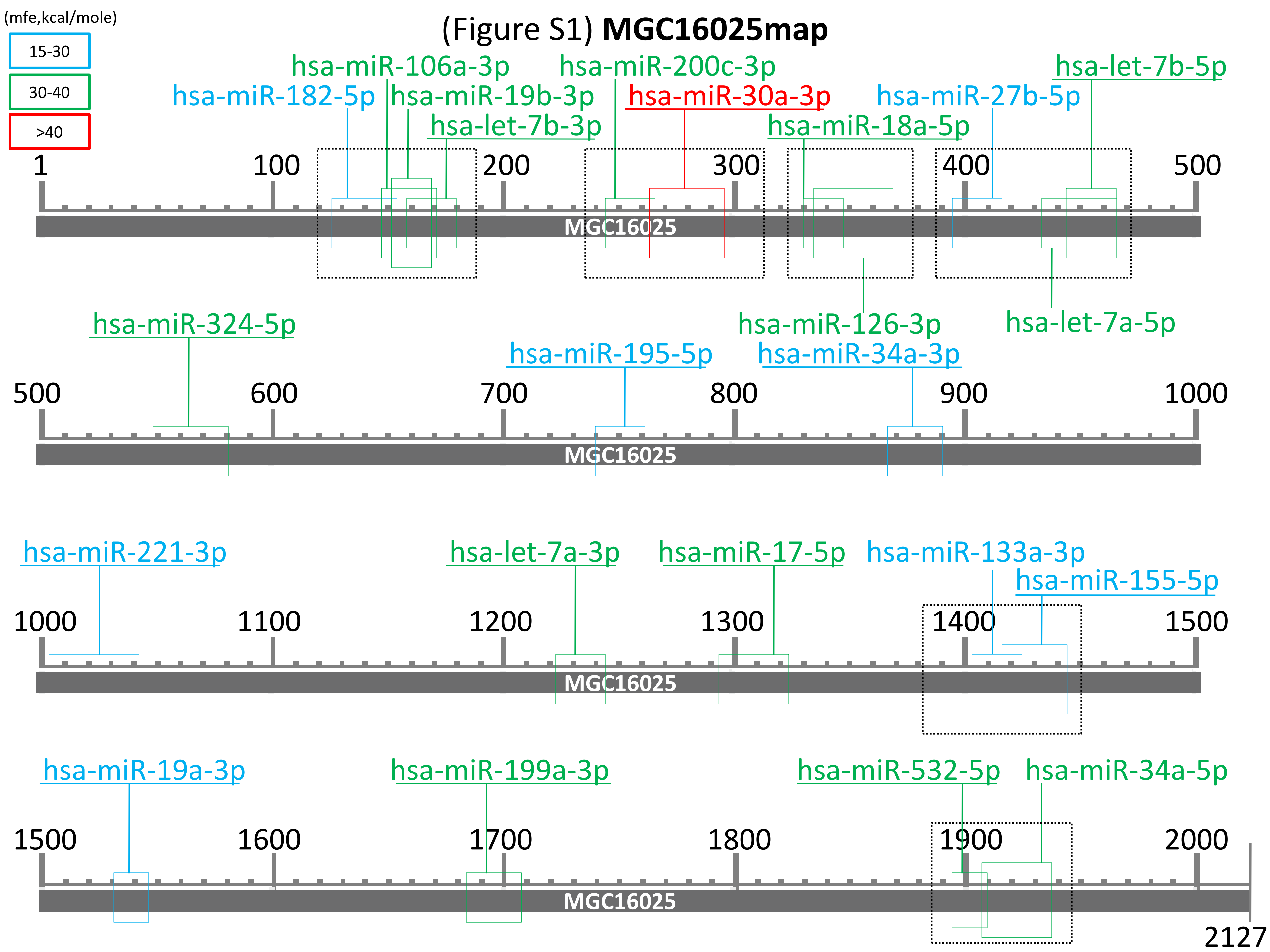

(Figure S2) **SPRY4-IT1map**

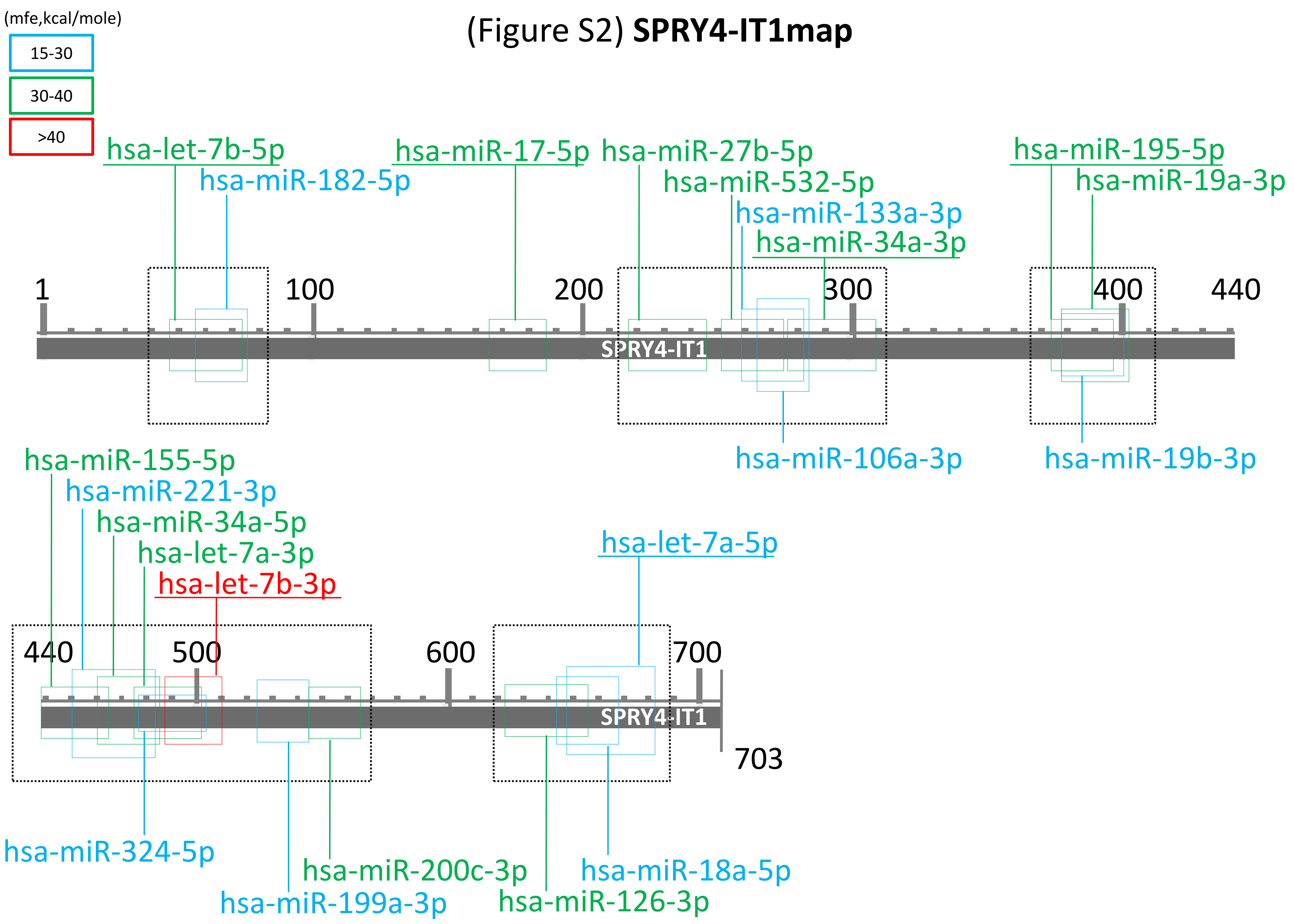

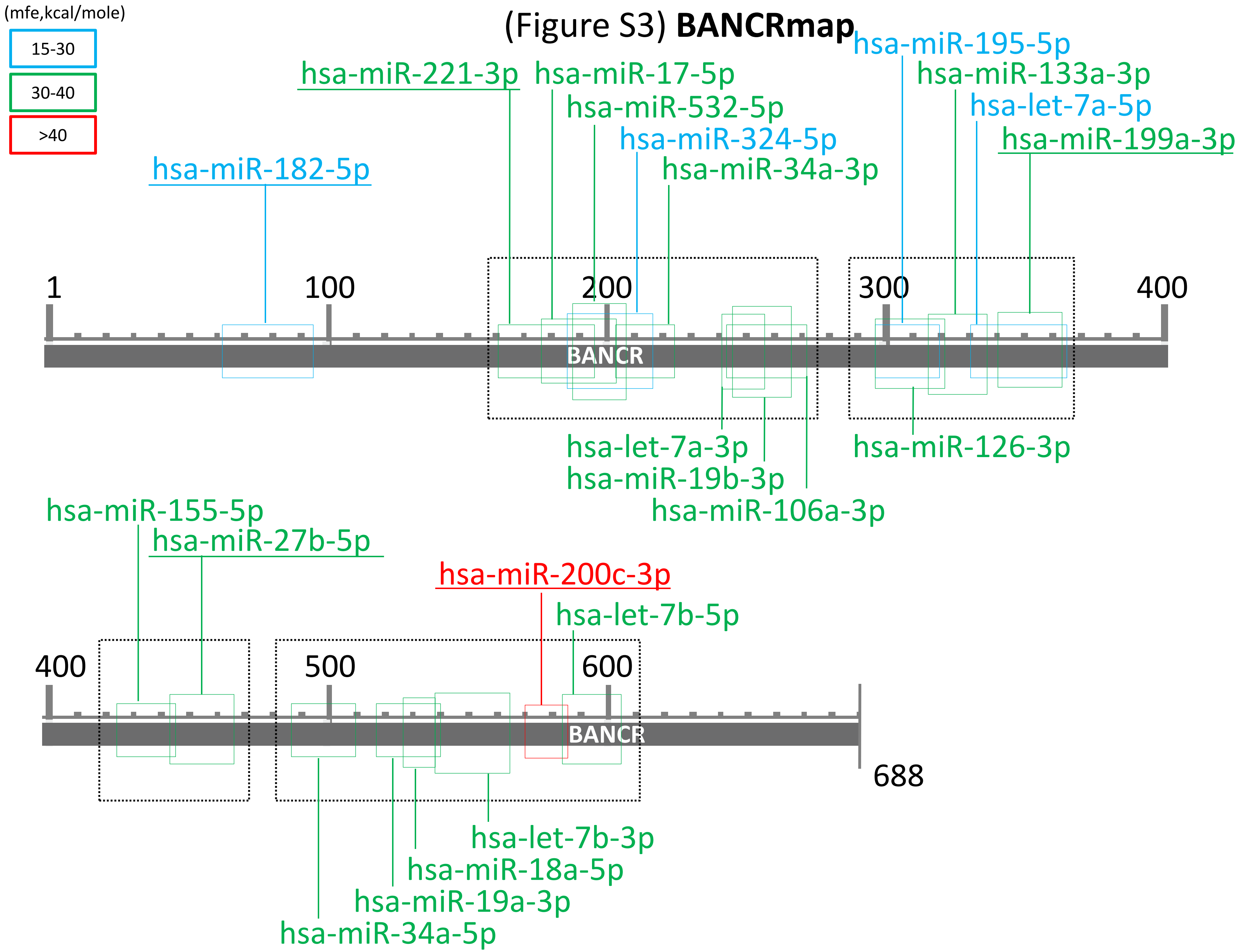

(mfe,kcal/mole)
15-30
30-40
>40
(Figure S3) BANCRmap
hsa-miR-195-5p
hsa-miR-221-3p
hsa-miR-17-5p
hsa-miR-133a-3p
hsa-miR-532-5p
hsa-let-7a-5p
hsa-miR-324-5p
hsa-miR-199a-3p
hsa-miR-182-5p
hsa-miR-34a-3p
1
100
200
300
400
BANCR
hsa-let-7a-3p
hsa-miR-126-3p
hsa-miR-19b-3p
hsa-miR-106a-3p
hsa-miR-155-5p
hsa-miR-27b-5p
hsa-miR-200c-3p
hsa-let-7b-5p
400
500
600
BANCR
688
hsa-let-7b-3p
hsa-miR-18a-5p
hsa-miR-19a-3p
hsa-miR-34a-5p

(Figure S4) SAMMSONmap

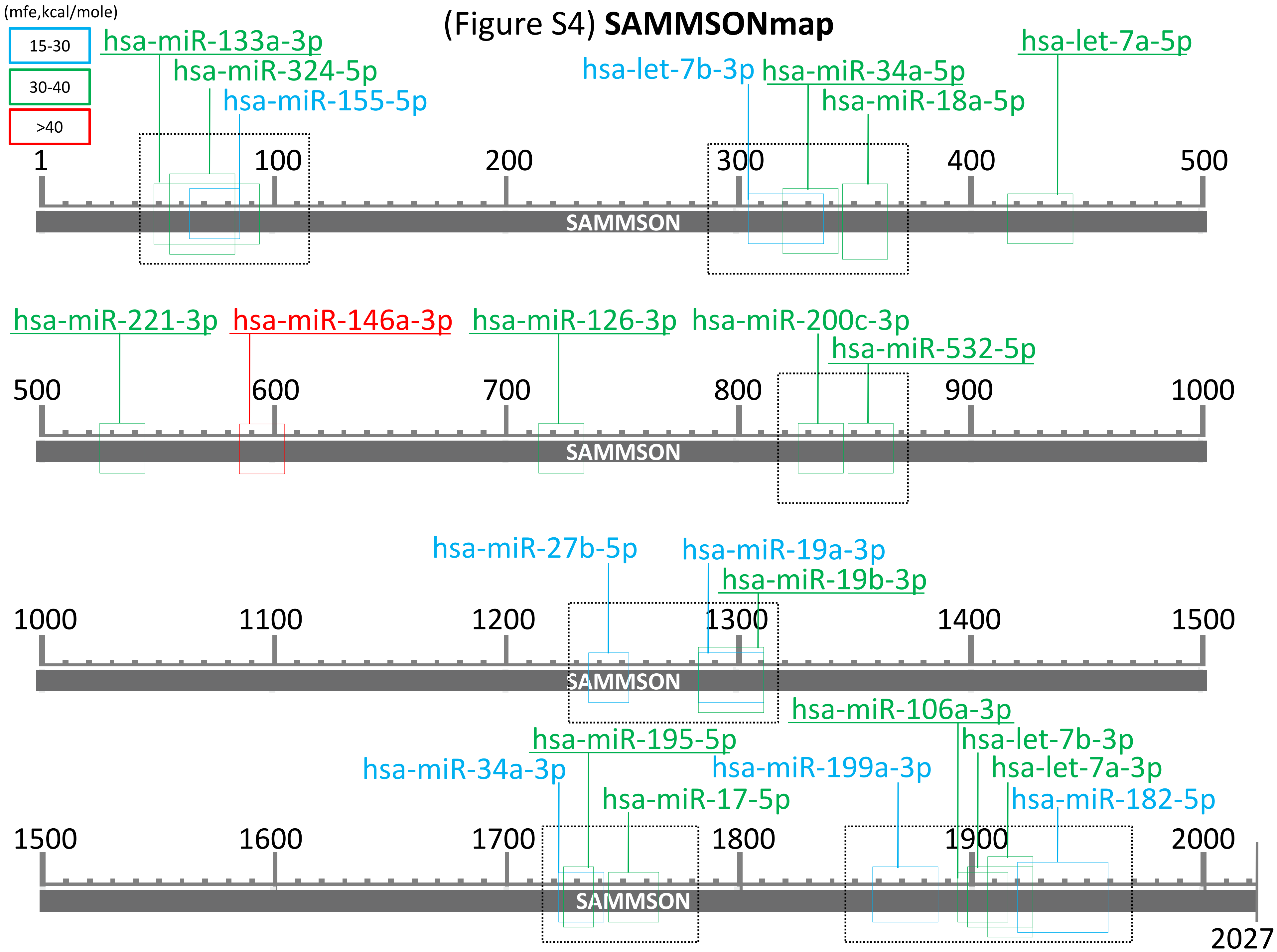

(Figure S5) **CDKN2B-AS1map**

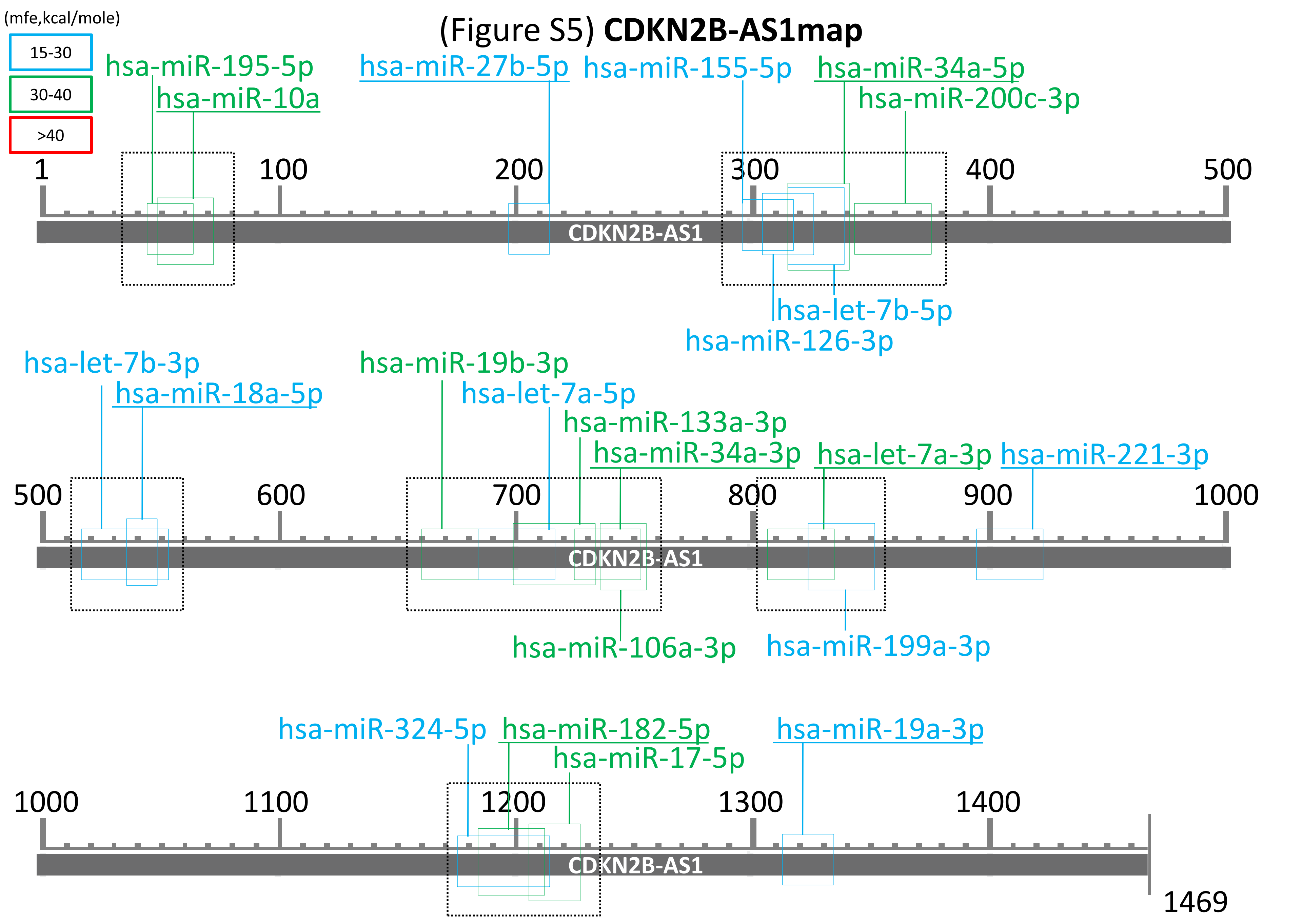

(Figure S6) **LINC00673map**

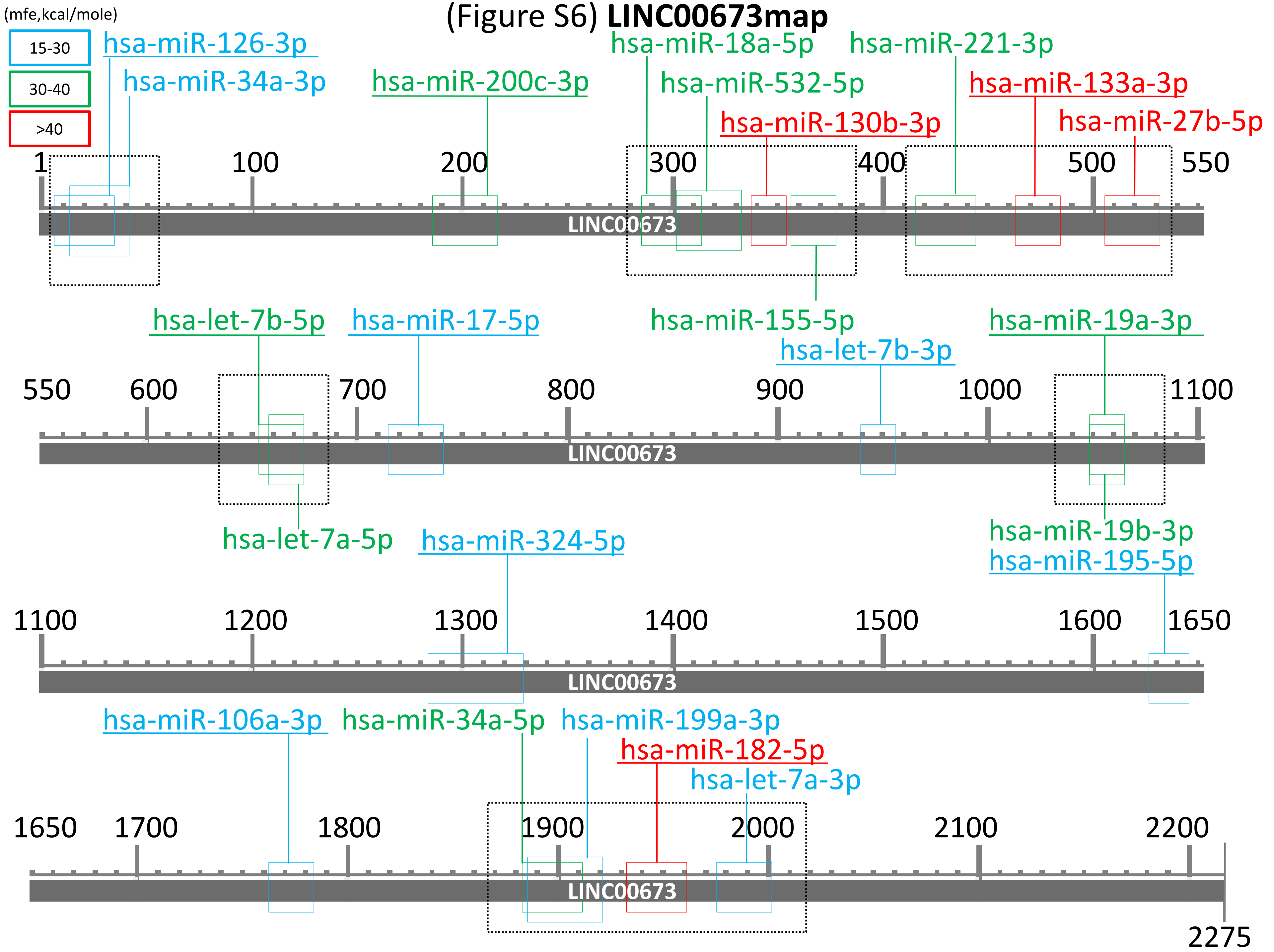

(Figure S7) **MIR31HGmap**

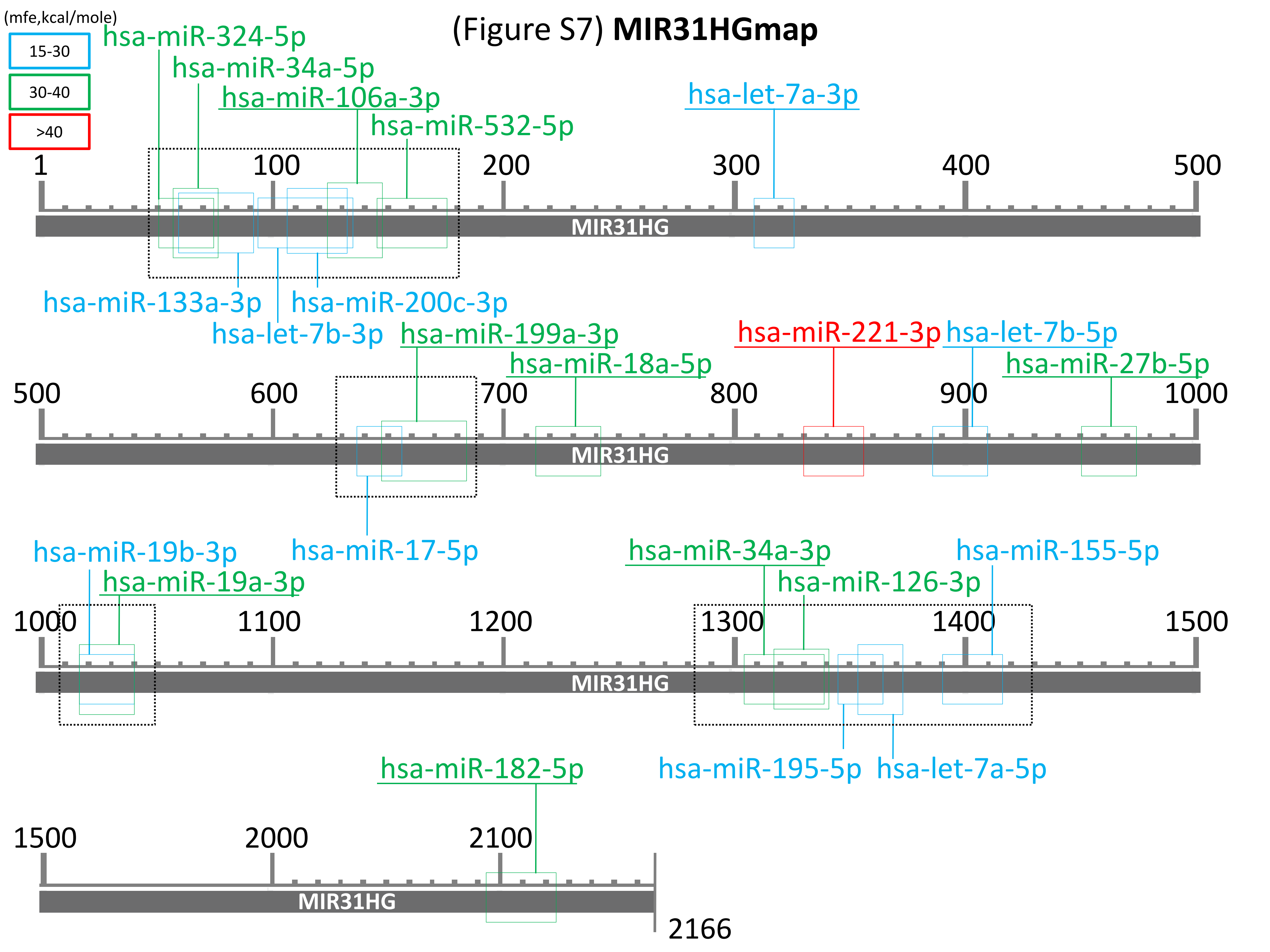

(Figure S8) **CASC15map**

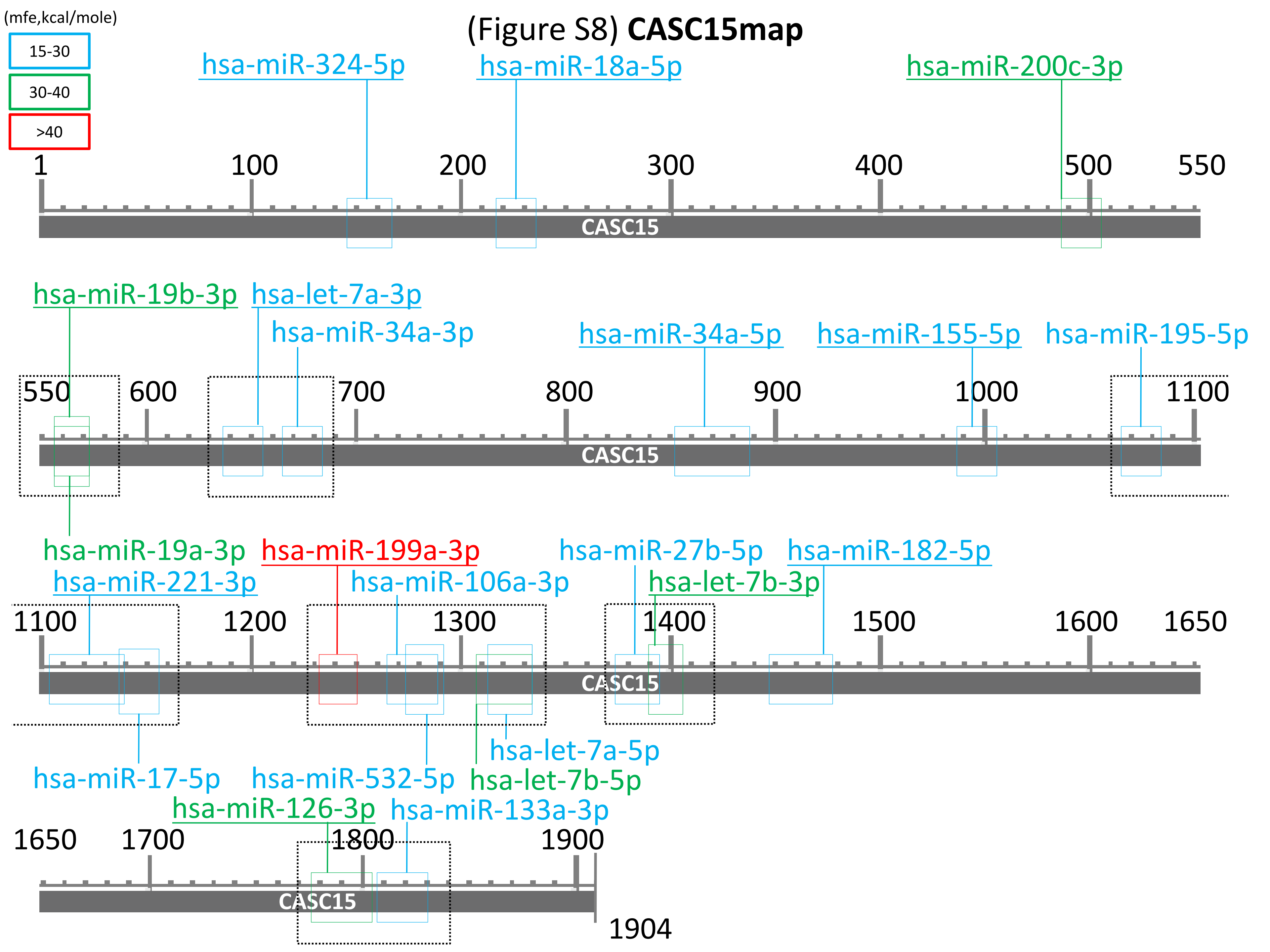

(Figure S9) **SNHG5map**

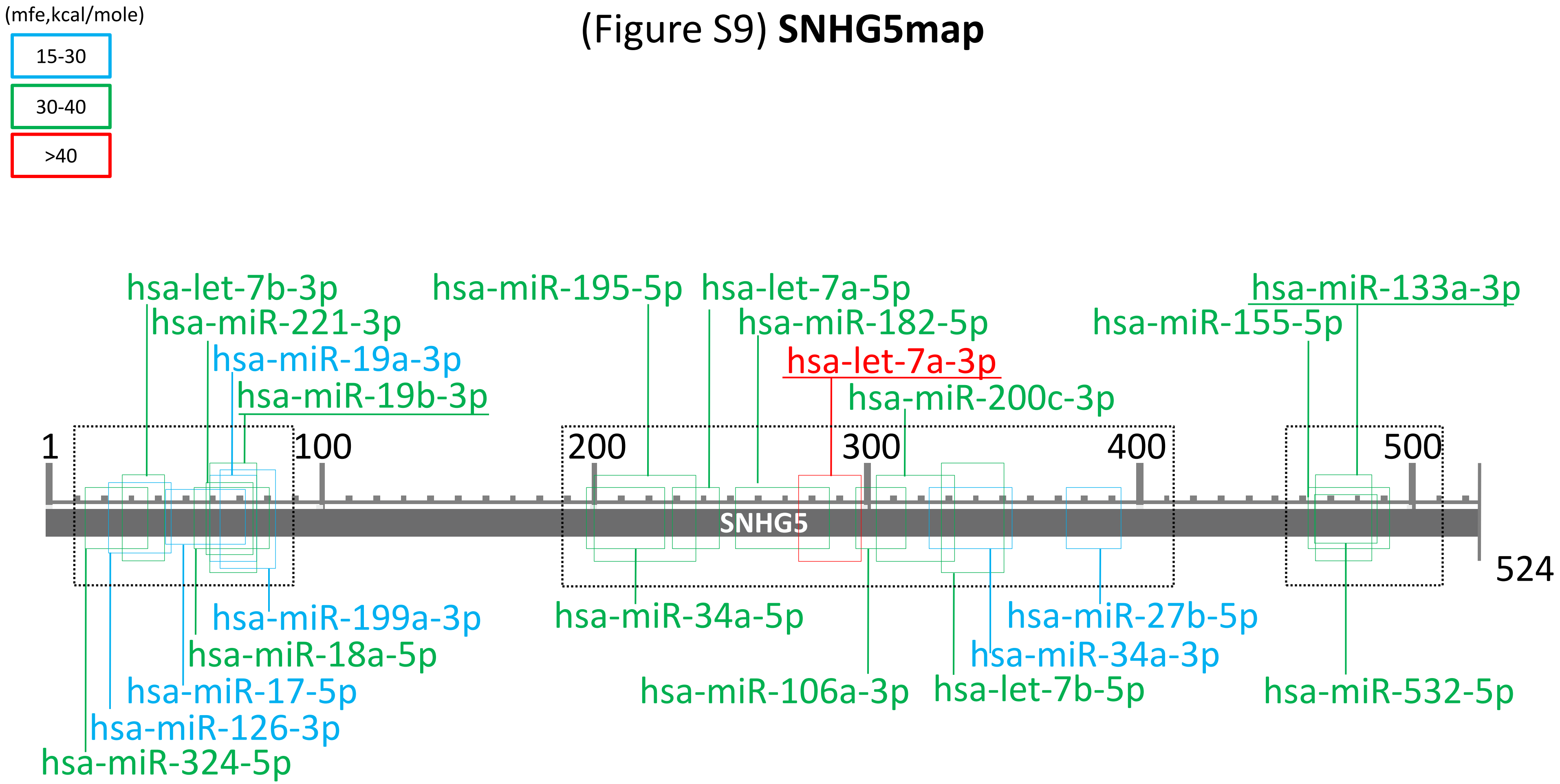

(Figure S10) **UCA1map**

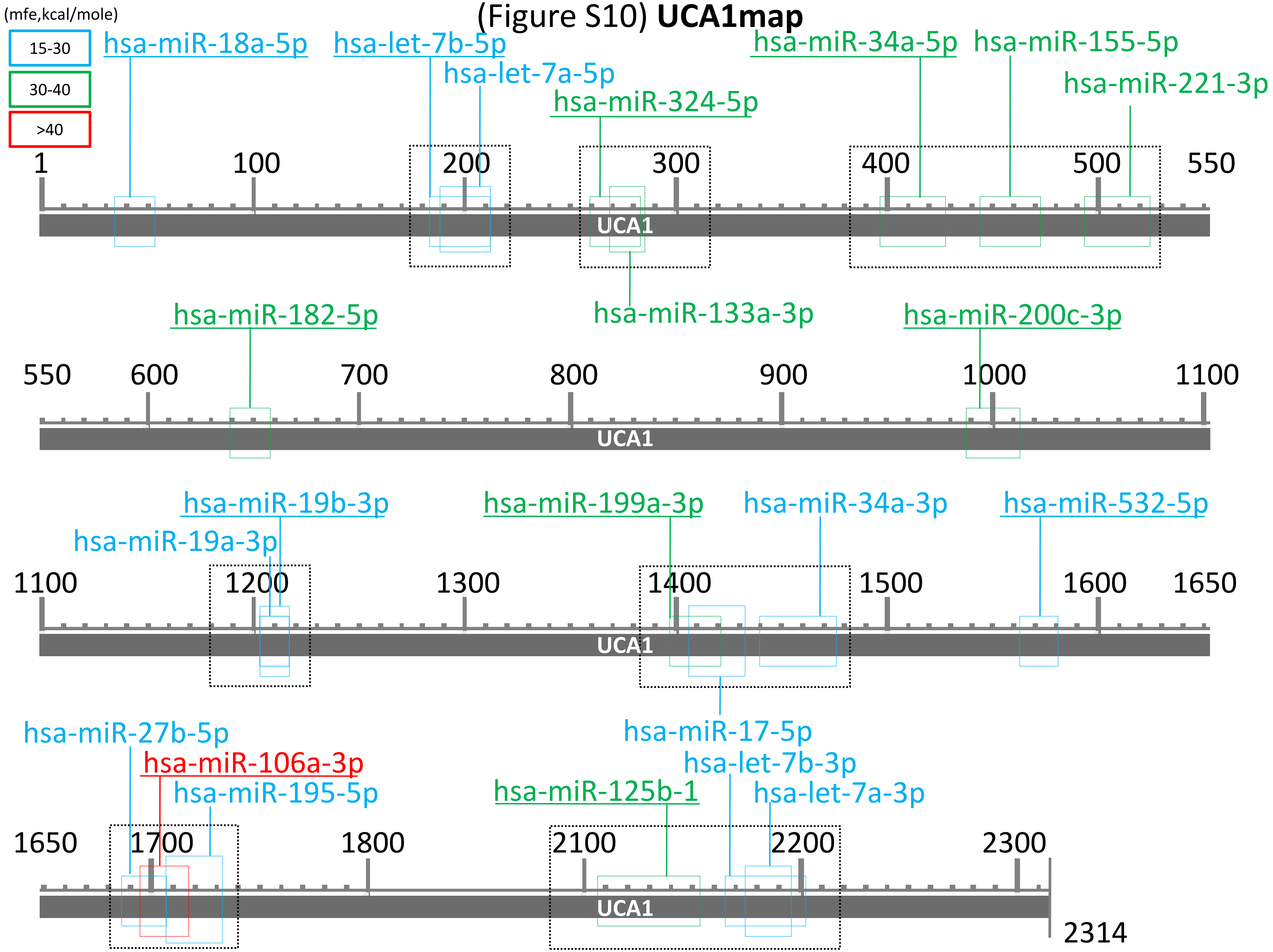

(Figure S11) HOTAIRmap

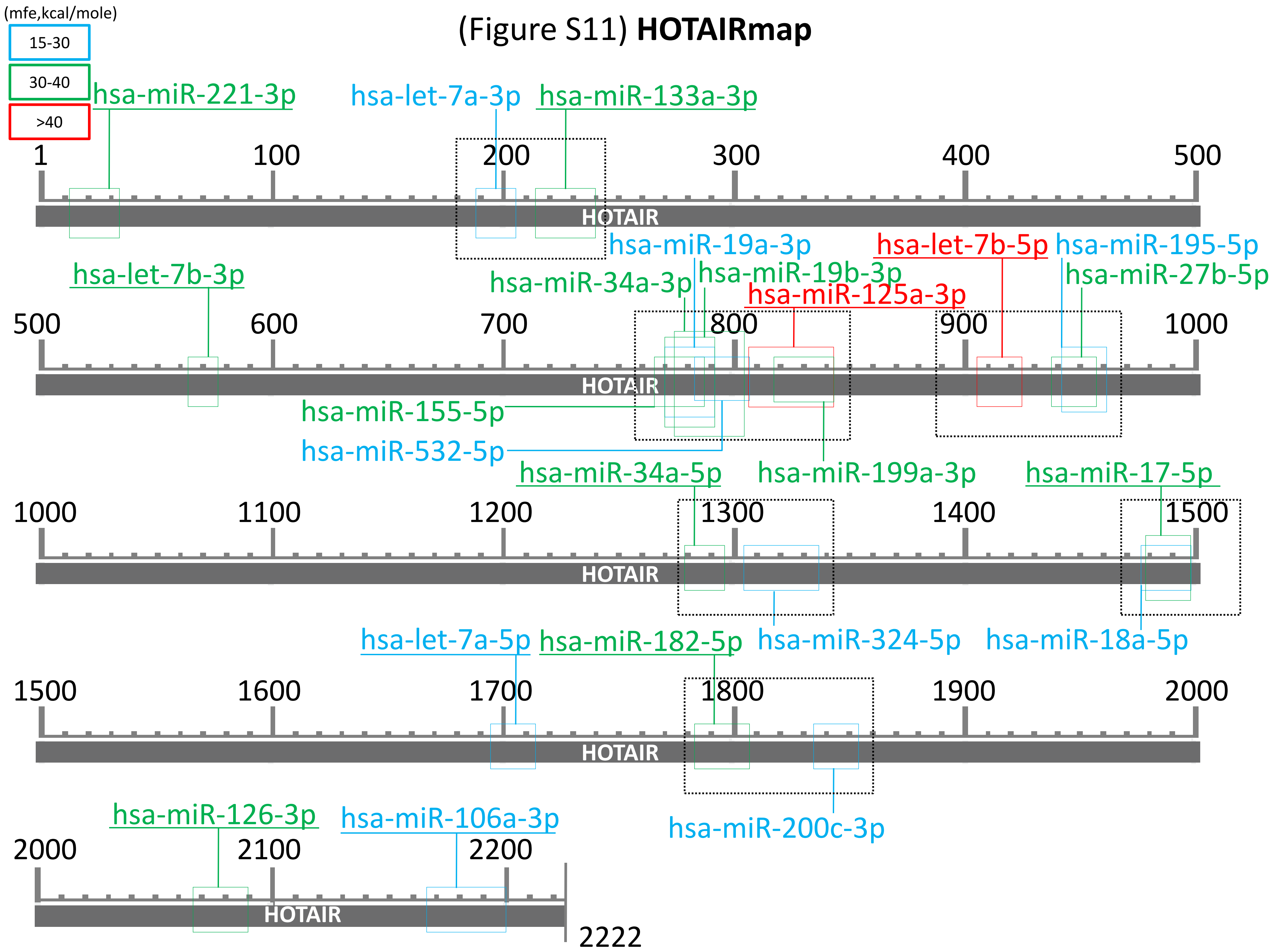

(Figure S12) **PTENP1map**

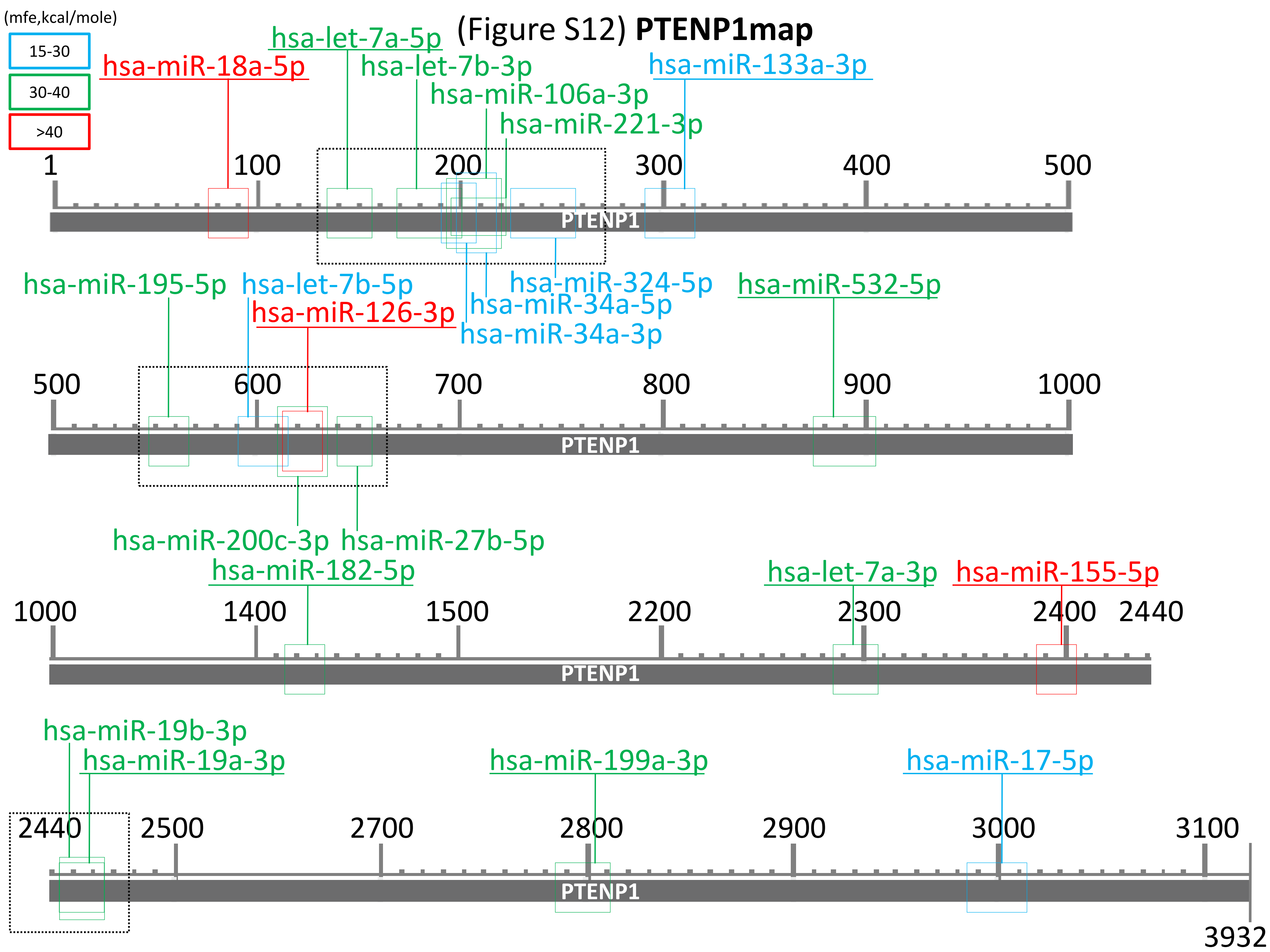

(Figure S13) **TUG1map**

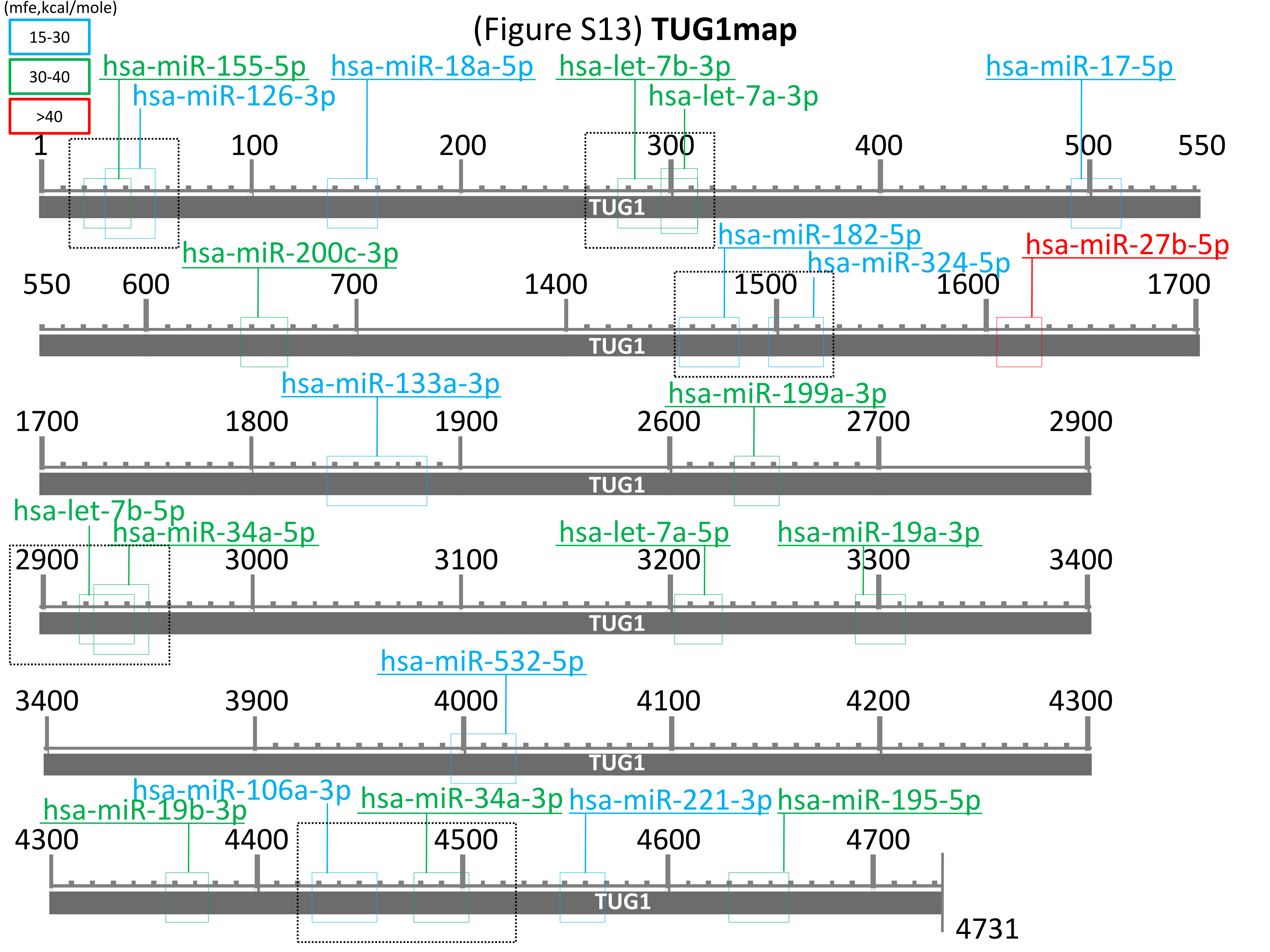

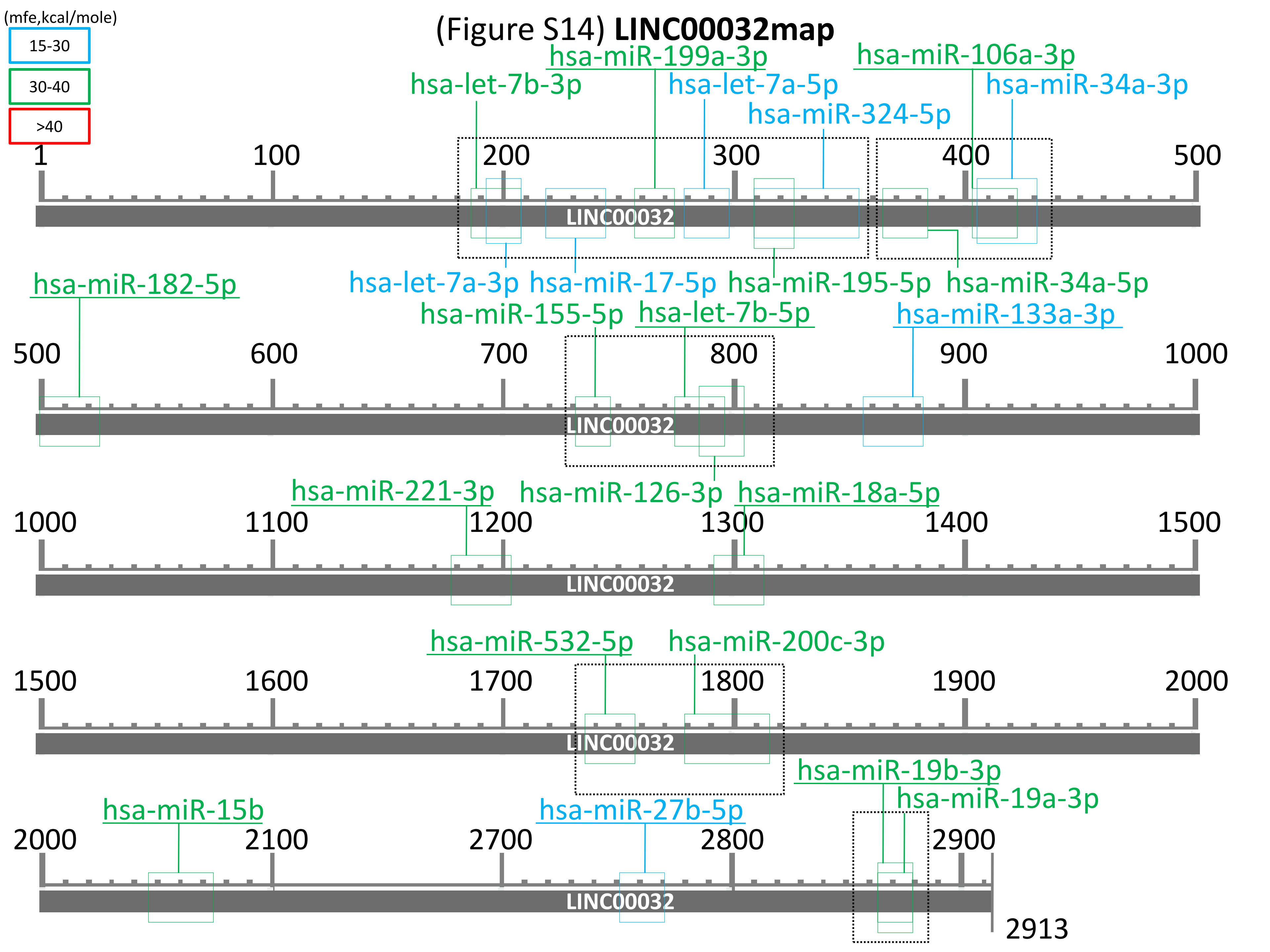
(mfe,kcal/mole)
15-30
30-40
>40
(Figure S14) LINC00032map
hsa-let-7b-3p
hsa-miR-199a-3p
hsa-let-7a-5p
hsa-miR-324-5p
hsa-miR-106a-3p
hsa-miR-34a-3p
1
100
200
300
400
500
LINC00032
hsa-let-7a-3p
hsa-miR-17-5p
hsa-miR-195-5p
hsa-miR-34a-5p
hsa-miR-182-5p
hsa-miR-155-5p
hsa-let-7b-5p
hsa-miR-133a-3p
500
600
700
800
900
1000
LINC00032
hsa-miR-221-3p
hsa-miR-126-3p
hsa-miR-18a-5p
1000
1100
1200
1300
1400
1500
LINC00032
hsa-miR-532-5p
hsa-miR-200c-3p
1500
1600
1700
1800
1900
2000
LINC00032
hsa-miR-19b-3p
hsa-miR-19a-3p
hsa-miR-15b
hsa-miR-27b-5p
2000
2100
2700
2800
2900
LINC00032
2913

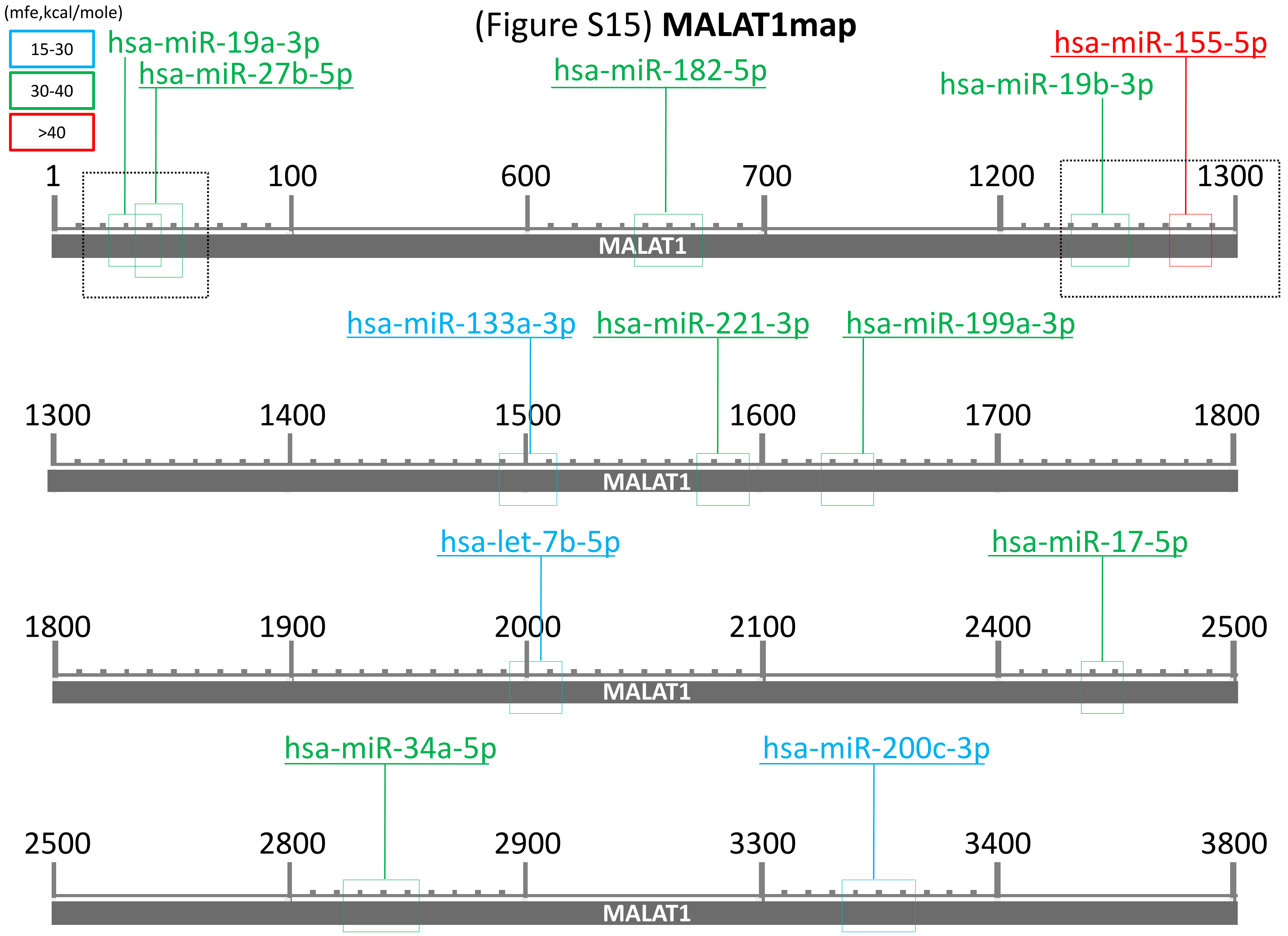

(Figure S15) MALAT1map
(mfe,kcal/mole)
15-30
30-40
>40
hsa-miR-19a-3p
hsa-miR-27b-5p
hsa-miR-182-5p
hsa-miR-155-5p
hsa-miR-19b-3p
1
100
600
700
1200
1300
MALAT1
hsa-miR-133a-3p
hsa-miR-221-3p
hsa-miR-199a-3p
1300
1400
1500
1600
1700
1800
MALAT1
hsa-let-7b-5p
hsa-miR-17-5p
1800
1900
2000
2100
2400
2500
MALAT1
hsa-miR-34a-5p
hsa-miR-200c-3p
2500
2800
2900
3300
3400
3800
MALAT1

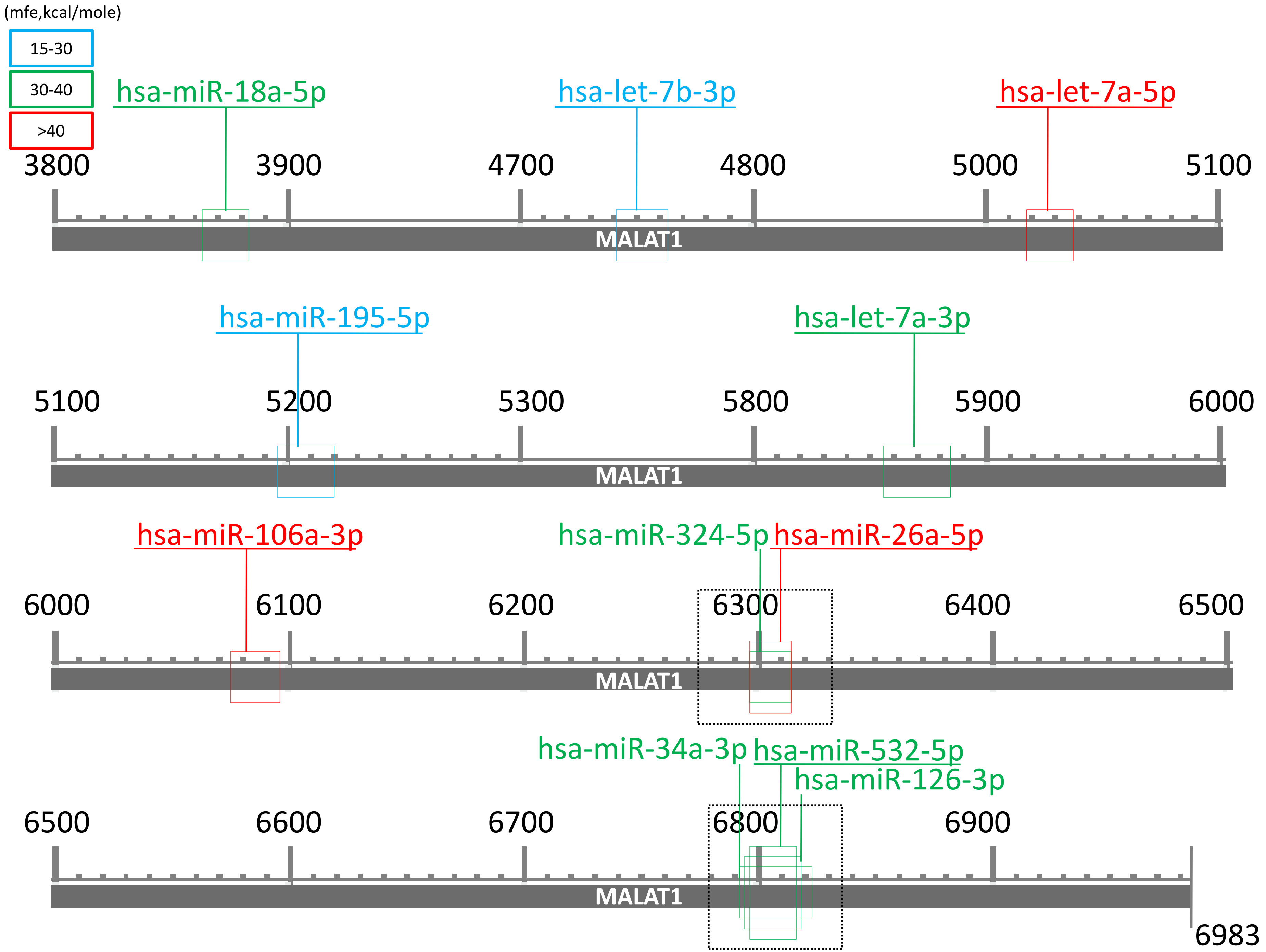

(mfe,kcal/mole)
15-30
30-40
>40
hsa-miR-18a-5p
hsa-let-7b-3p
hsa-let-7a-5p
3800
3900
4700
4800
5000
5100
MALAT1
hsa-miR-195-5p
hsa-let-7a-3p
5100
5200
5300
5800
5900
6000
MALAT1
hsa-miR-106a-3p
hsa-miR-324-5p
hsa-miR-26a-5p
6000
6100
6200
6300
6400
6500
MALAT1
hsa-miR-34a-3p
hsa-miR-532-5p
hsa-miR-126-3p
6500
6600
6700
6800
6900
MALAT1
6983

(Figure S16) **GAS5map**

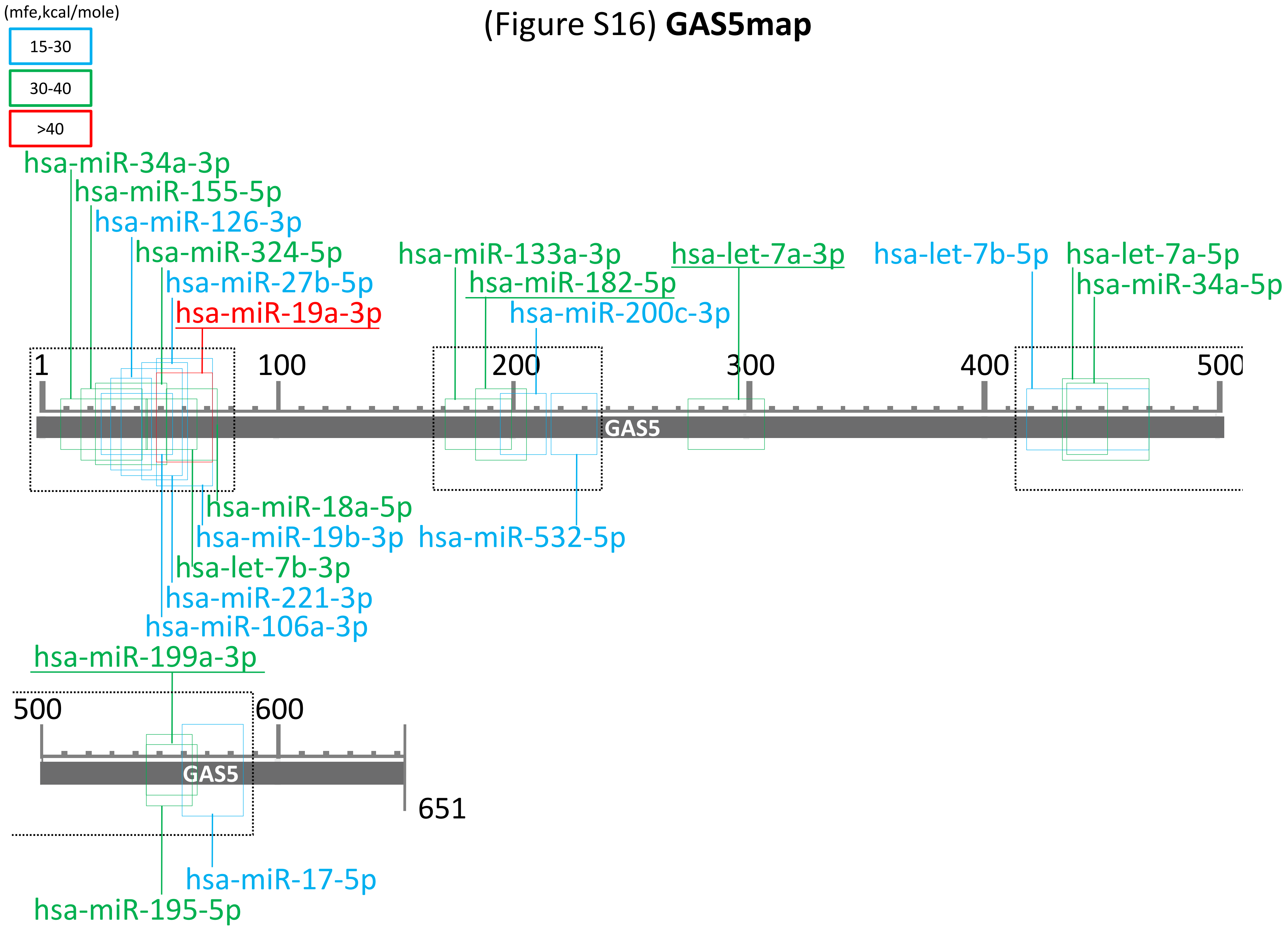

(Figure S17) **H19map**

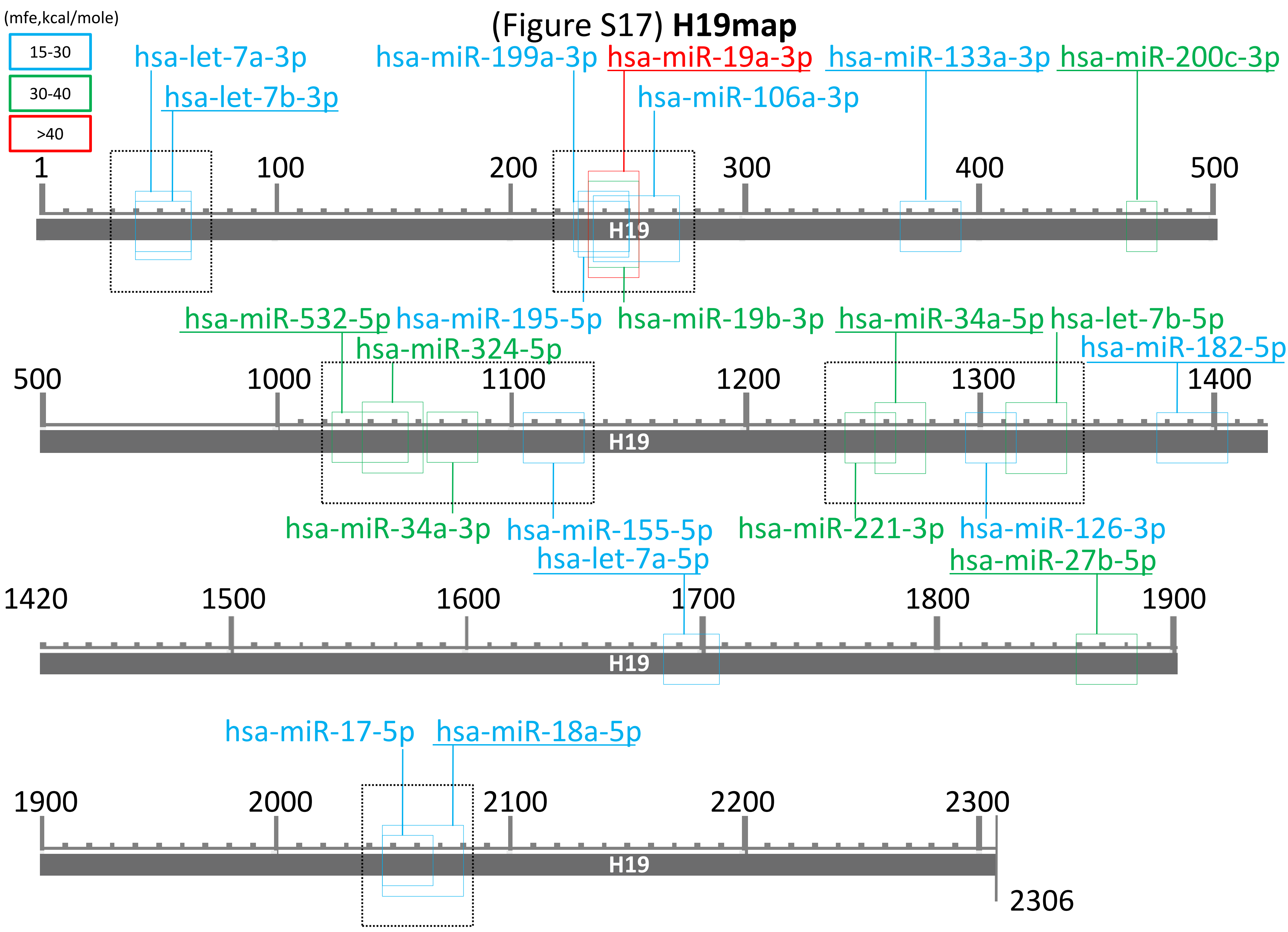

Supplement: Supplementary file 1 — Additional file 1: Table S1A. Predicted lncRNA-miRNA interactions; Table S1B. MiRNA-target gene interactions; Table S1C. TF-miRNA interactions; Table S1D. Predicted lncRNA-TF interactions; Table S1E. TF-TF interactions; Table S1F. Topological and non-topological parameters calculated for each node of regulatory network motif; Table S1G. Prioritized motifs for metastatic and non-metastatic melanoma phenotype; Table S1H. Predictive statistics for motif 1; Table S1I. Predictive statistics for motif 2; Table S1J. Predictive statistics for motif 3; Table S1K.P-value identified from pairwise and overall comparison of three patient subgroups; Table S2. Weighting scenarios for ranking of motifs; Table S3. Patient-derived RNAseq expression profile (pan-cancer normalized log 2) of nodes in three prioritized motifs (lncRNA/miRNA/TF); Figures S1-S17. Hybridization maps of putative miRNAs binding sites across lncRNA sequences; Figure S18. LncRNA-miRNA interaction network. Rectangular nodes designate lncRNA (peach color) and miRNA (cyan color). The network consists of 47 nodes (including 17 lncRNAs and 30 miRNAs) and 174 prioritized edges link the pairs of lncRNA and miRNAs in cluster; Figure S19. TF-miRNA interaction network. Experimentally validated target genes of miRNAs which act as TFs are represented by octagon nodes (yellow color) and miRNAs are showed by rectangular nodes (cyan color). The network is comprised of 146 nodes with 25 TFs and 121 miRNAs. The edges of the network (total 247) signify predictions of miRNA regulation by TFs. Arrow-headed lines are for activation (purple color) and bar-headed lines are for repression (green color); Data S1. Python script for retrieval of FASTA sequences from NCBI; Data S2. Pseudo code for ranking of network motifs. [file 12859_2020_3656_MOESM1_ESM.zip › Figure S1-S17.pdf]
