# Supplementary material for: An integrative network-driven pipeline for systematic identification of lncRNA-associated regulatory network motifs in metastatic melanoma
Source: BMC Bioinformatics. 2020 Jul 23;21:329. doi: 10.1186/s12859-020-03656-6 (PMC7376740; doi:10.1186/s12859-020-03656-6)
Supplement: Supplementary file 1 — Additional file 1: Table S1A. Predicted lncRNA-miRNA interactions; Table S1B. MiRNA-target gene interactions; Table S1C. TF-miRNA interactions; Table S1D. Predicted lncRNA-TF interactions; Table S1E. TF-TF interactions; Table S1F. Topological and non-topological parameters calculated for each node of regulatory network motif; Table S1G. Prioritized motifs for metastatic and non-metastatic melanoma phenotype; Table S1H. Predictive statistics for motif 1; Table S1I. Predictive statistics for motif 2; Table S1J. Predictive statistics for motif 3; Table S1K.P-value identified from pairwise and overall comparison of three patient subgroups; Table S2. Weighting scenarios for ranking of motifs; Table S3. Patient-derived RNAseq expression profile (pan-cancer normalized log 2) of nodes in three prioritized motifs (lncRNA/miRNA/TF); Figures S1-S17. Hybridization maps of putative miRNAs binding sites across lncRNA sequences; Figure S18. LncRNA-miRNA interaction network. Rectangular nodes designate lncRNA (peach color) and miRNA (cyan color). The network consists of 47 nodes (including 17 lncRNAs and 30 miRNAs) and 174 prioritized edges link the pairs of lncRNA and miRNAs in cluster; Figure S19. TF-miRNA interaction network. Experimentally validated target genes of miRNAs which act as TFs are represented by octagon nodes (yellow color) and miRNAs are showed by rectangular nodes (cyan color). The network is comprised of 146 nodes with 25 TFs and 121 miRNAs. The edges of the network (total 247) signify predictions of miRNA regulation by TFs. Arrow-headed lines are for activation (purple color) and bar-headed lines are for repression (green color); Data S1. Python script for retrieval of FASTA sequences from NCBI; Data S2. Pseudo code for ranking of network motifs. [file Figure S18-S19.pdf › Figure S18-S19.pdf]

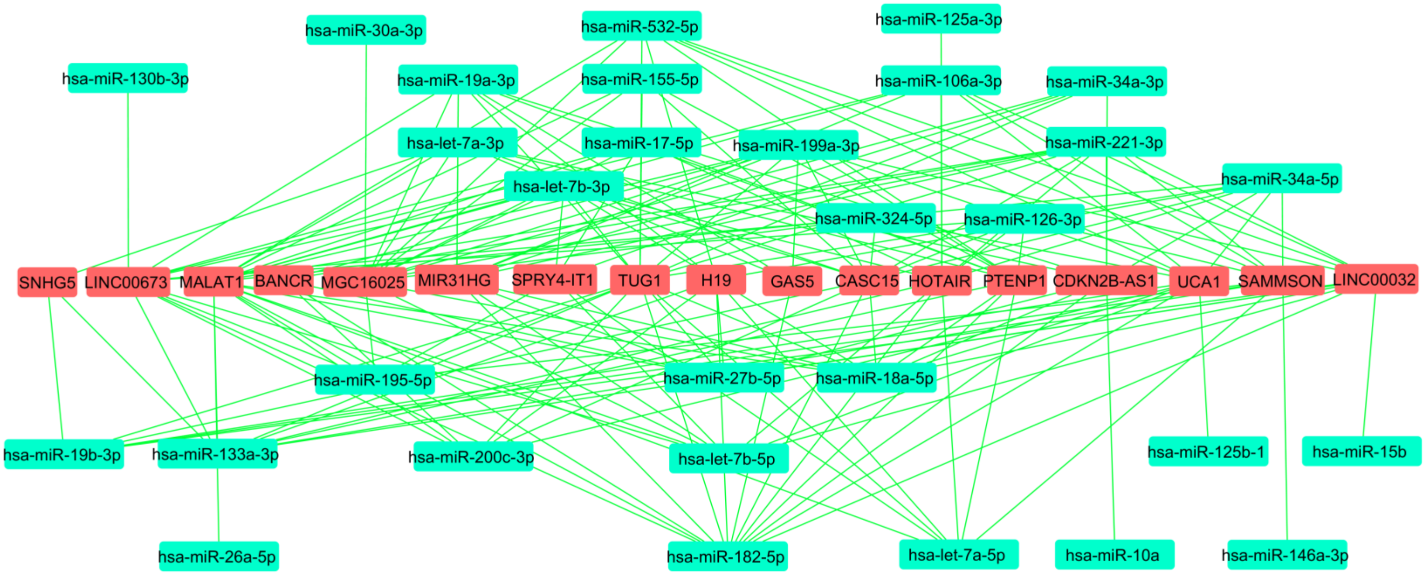

Figure S18: LncRNA-miRNA interaction network. Rectangular nodes designate lncRNA (peach color) and miRNA (cyan color). The network consists of 47 nodes (including 17 lncRNAs and 30 miRNAs) and 174 prioritized edges link the pairs of lncRNA and miRNAs in cluster. Abbreviations: LncRNA, long non-coding RNA; miRNA, microRNA.

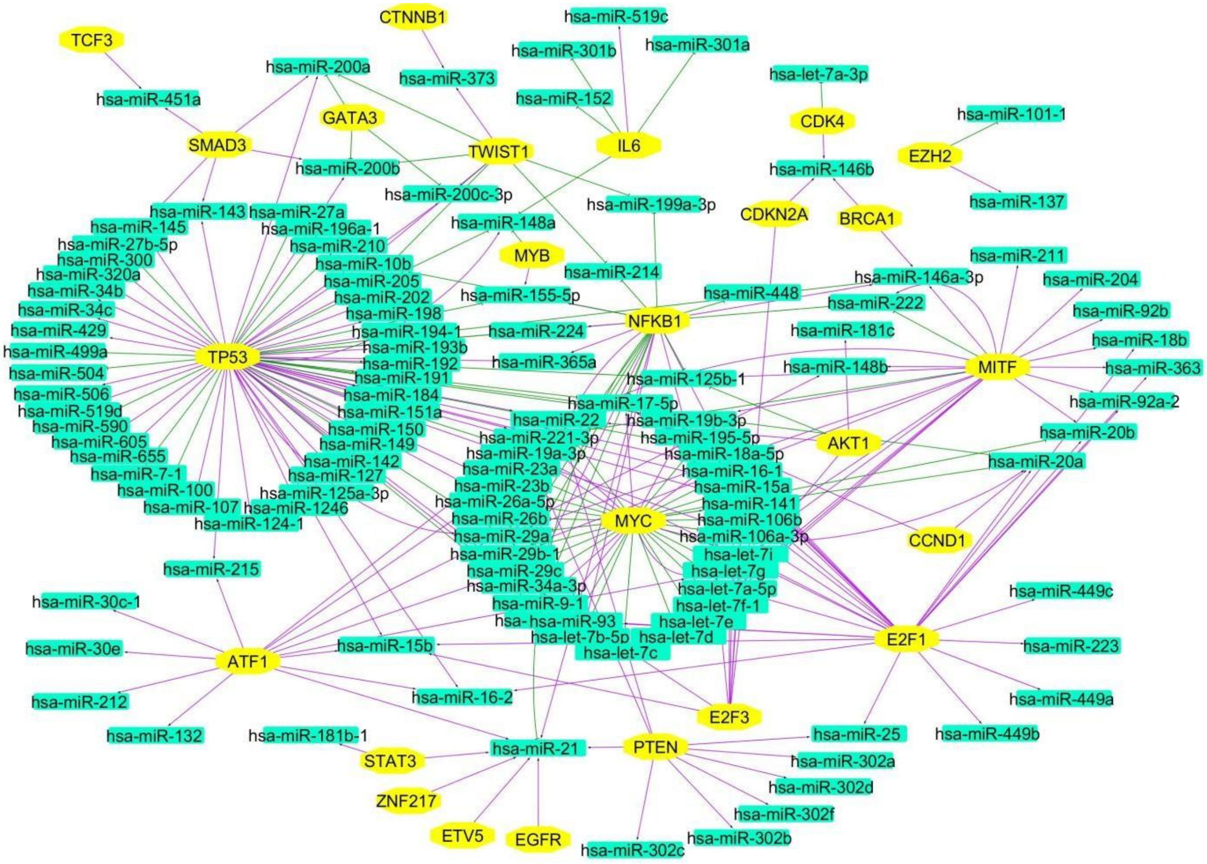

Figure S19: TF-miRNA interaction network. Experimentally validated target genes of miRNAs which act as TFs are represented by octagon nodes (yellow color) and miRNAs are showed by rectangular nodes (cyan color). The network comprised of 146 nodes with 25 TFs and 121 miRNAs. The edges of the network (total 247) signify prediction of miRNA regulation by TF. Arrow-headed lines are for activation (purple color) and bar-headed lines are for repression (green color). Abbreviation: TF, transcription factor; miRNA, microRNA.
